# Frequency of transmission, asymptomatic shedding, and airborne spread of *Streptococcus pyogenes* in schoolchildren exposed to scarlet fever: a prospective, longitudinal, multicohort, molecular epidemiological, contact-tracing study in England, UK

**DOI:** 10.1016/S2666-5247(21)00332-3

**Published:** 2022-05

**Authors:** Rebecca Cordery, Amrit K Purba, Lipi Begum, Ewurabena Mills, Mia Mosavie, Ana Vieira, Elita Jauneikaite, Rhoda C Y Leung, Matthew K Siggins, Derren Ready, Peter Hoffman, Theresa Lamagni, Shiranee Sriskandan

**Affiliations:** a London Health Protection Teams, Public Health England, London, UK; b National Infection Service, Public Health England, London, UK; c Department of Infectious Disease, Imperial College London, London, UK; d NIHR Health Protection Research Unit in Healthcare Associated Infections and Antimicrobial Resistance, Imperial College London, London, UK; e MRC Centre for Molecular Bacteriology and Infection, Imperial College London, London, UK; f Department of Infectious Disease Epidemiology, School of Public Health, Imperial College London, London, UK; g NIHR Health Protection Research Unit in Behavioural Science and Evaluation, University of Bristol, Bristol, UK

## Abstract

**Background:**

Despite recommendations regarding prompt treatment of cases and enhanced hygiene measures, scarlet fever outbreaks increased in England between 2014 and 2018. We aimed to assess the effects of standard interventions on transmission of *Streptococcus pyogenes* to classroom contacts, households, and classroom environments to inform future guidance.

**Methods:**

We did a prospective, longitudinal, multicohort, molecular epidemiological, contact-tracing study in six settings across five schools in Greater London, UK. Schools and nurseries were eligible to participate if they had reported two cases of scarlet fever within 10 days of each other among children aged 2–8 years from the same class, with the most recent case arising in the preceding 48 h. We cultured throat swabs from children with scarlet fever, classroom contacts, and household contacts at four timepoints. We also cultured hand swabs and cough plates from all cases in years 1 and 2 of the study, and from classroom contacts in year 2. Surface swabs from toys and other fomites in classrooms were cultured in year 1, and settle plates from classrooms were collected in year 2. Any sample with *S pyogenes* detected was recorded as positive and underwent *emm* genotyping and genome sequencing to compare with the outbreak strain.

**Findings:**

Six classes, comprising 12 cases of scarlet fever, 17 household contacts, and 278 classroom contacts were recruited between March 1 and May 31, 2018 (year 1), and between March 1 and May 31, 2019 (year 2). Asymptomatic throat carriage of the outbreak strains increased from 11 (10%) of 115 swabbed children in week 1, to 34 (27%) of 126 in week 2, to 26 (24%) of 108 in week 3, and then five (14%) of 35 in week 4. Compared with carriage of outbreak *S pyogenes* strains, colonisation with non-outbreak and non-genotyped *S pyogenes* strains occurred in two (2%) of 115 swabbed children in week 1, five (4%) of 126 in week 2, six (6%) of 108 in week 3, and in none of the 35 children in week 4 (median carriage for entire study 2·8% [IQR 0·0–6·6]). Genome sequencing showed clonality of outbreak isolates within each of six classes, confirming that recent transmission accounted for high carriage. When transmissibility was tested, one (9%) of 11 asymptomatic carriers of *e*mm4 and five (36%) of 14 asymptomatic carriers of *emm3.93* had a positive cough plate. The outbreak strain was identified in only one (2%) of 60 surface swabs taken from three classrooms; however, in the two classrooms with settle plates placed in elevated locations, two (17%) of 12 and six (50%) of 12 settle plates yielded the outbreak strain.

**Interpretation:**

Transmission of *S pyogenes* in schools is intense and might occur before or despite reported treatment of cases, underlining a need for rapid case management. Despite guideline adherence, heavy shedding of *S pyogenes* by few classroom contacts might perpetuate outbreaks, and airborne transmission has a plausible role in its spread. These findings highlight the need for research to improve understanding and to assess effectiveness of interventions to reduce airborne transmission of *S pyogenes*.

**Funding:**

Action Medical Research, UK Research Innovation, and National Institute for Health Research.

## Introduction

Since 2014, England has seen an upsurge in the prevalence of scarlet fever that is unprecedented in modern times.^1^, ^2^ Scarlet fever is a highly communicable exanthem caused by *Streptococcus pyogenes* (group A streptococcus) that predominantly affects younger children (aged 4–7 years). In England and Wales, more than 30 000 cases of scarlet fever were reported in 2018, the highest number since 1960, with an age-specific incidence of 523 cases per 100 000 children aged 1–4 years.^3^ Infections have long been recognised to cause outbreaks in schools and nurseries, creating a substantial public health burden.^2^ Although benign if treated promptly, individuals living in a household with a recent case of scarlet fever have a 20-times increased risk of invasive streptococcal infection.^4^ In 2016, a near two-times increase in notifications of invasive disease was accompanied by the emergence of a new sublineage of *emm1 S pyogenes* (M1_UK_), which expresses increased amounts of scarlet fever toxin.^5^

**Research in context**

**Evidence before this study**

Since 2014, an unprecedented upsurge in the prevalence of scarlet fever, an infectious exanthem triggered by superantigen toxin-expressing *Streptococcus pyogenes*, has been reported among children in England (UK). Despite national guidance regarding prompt treatment of cases and the importance of hand hygiene, notifications of cases and outbreaks in educational settings increased further in 2018. Increased prevalence of scarlet fever not only creates a considerable individual and public health burden, but is also associated with an increased incidence of *S pyogenes* throat infections and more lethal invasive infections in both children and adults. Older studies have provided evidence of fomite and airborne dispersal of *S pyogenes*; however, they do not provide evidence of strain-specific transmission or relate to modern contextual settings. To identify potential interventions, on July 4, 2021, we searched PubMed for contemporary descriptions of the mode of scarlet fever transmission, using the terms “scarlet fever”, “outbreak”, “school”, and “transmission”, without language restrictions. We also considered related articles in PubMed or referenced articles. Four studies reported findings of single scarlet fever outbreaks, in which the attack rate was high. All studies reported a high prevalence of *S pyogenes* infection among contacts at a single timepoint, although none undertook detailed studies of the mechanism of transmission.

**Added value of this study**

In this prospective, longitudinal, multicohort, molecular epidemiological, contact-tracing study in six educational settings, we found that transmission of *S pyogenes* was intense in classroom settings with two recent cases of scarlet fever, even when attack rates were modest. Genome sequencing confirmed that a unique outbreak strain infected over a quarter of the children in affected classes. In some settings, almost half of all children carried the outbreak strain at some point, whereas carriage of non-outbreak strains was uncommon. As such, asymptomatic carriage rates of *S pyogenes* should be interpreted with caution, using contextual information regarding season and outbreaks. Despite treatment and temporary exclusion of children with scarlet fever, as well as hygiene interventions that reduced risk of fomite contamination, classroom contacts were often already infected with outbreak strains. Most children with infection were asymptomatic; however, a quarter of cases showed bacterial shedding suggestive of bacterial replication and potential infectiousness. Furthermore, we found that colonisation rates increased in classmates over time and that outbreak strains of *S pyogenes* dispersed into the air of the classrooms tested with settle plates. Conducted at a time just before the COVID-19 pandemic, the study provides insight into the spread of respiratory infection without physical distancing.

**Implications of all the available evidence**

The transmissibility of *S pyogenes* within school settings exceeds that of many other major pathogens and might feature so-called super spreaders. Rapid intervention is required to identify, isolate, and treat cases of scarlet fever; to reduce the initial infective burden in classroom settings; and to prevent outbreaks. Given that the strains that cause scarlet fever also cause *S pyogenes* pharyngitis, consideration should be given to managing scarlet fever and *S pyogenes* pharyngitis as single entities in young children. Altered diagnostic and management algorithms adapted to young children might be required. Within schools, physical distancing, improved hygiene measures, and increased ventilation could have a major role in interrupting transmission, as evidenced by considerable curtailment of scarlet fever notifications during COVID-19-related lockdowns. Additionally, our findings should inform future responses to other types of *S pyogenes* outbreaks, by underlining a possible role for airborne transmission that might prompt environmental air sampling, identification of so-called heavy shedders, and a need to research potential use of surgical masks for patients at risk of invasive *S pyogenes* infection.

The literature highlights a role for fomite surfaces in the propagation of *S pyogenes* outbreaks.^6^, ^7^ Guidance for management of school or nursery outbreaks of scarlet fever emphasises the importance of maintaining good hand hygiene, prompt antibiotic treatment of index cases, and exclusion from school or nursery until 24 h after antibiotic treatment has started.^8^ Escalation of interventions, including daily cleaning of toys, utensils, and frequent touchpoints, is recommended if necessary, along with daily vacuuming of soft furnishings.^8^

To understand why outbreaks might continue to occur despite following these interventions, we investigated transmission of *S pyogenes* during the scarlet fever seasons of 2018 and 2019, focusing on educational settings with two sequential notifications of scarlet fever. We primarily aimed to establish the effects of standard interventions from existing guidance, which focus on hygiene and case exclusion, on transmission of *S pyogenes* to children, classroom contacts, households, and classroom environments. Additionally, we aimed to investigate modes of transmission to inform future guidance and to provide insight into outbreaks of *S pyogenes* more generally.

## Methods

### Study design and participants

We did a prospective, longitudinal, multicohort, molecular epidemiological, contact-tracing study of scarlet fever in primary school aged children (aged 2–8 years) in six settings in Greater London, UK. We recruited schools and nurseries during the scarlet fever seasons of 2 consecutive years: from March 1 to May 31, 2018 (year 1), and from March 1 to May 31, 2019 (year 2).

Schools and nurseries from the Greater London area that notified scarlet fever cases to local Health Protection teams were invited to participate if they reported two confirmed or probable cases of scarlet fever within 10 days of each other among children aged 2–8 years from the same class, with the most recent case arising in the preceding 48 h (appendix pp 2, 19). Routine public health advice was provided, including advice that children with infection should be excluded from school until they had received at least 24 h of antibiotic treatment.^8^ Based on a pragmatic approach, the first location with two eligible cases that agreed to participate each month was selected.

The study protocol was approved by a Research Ethics Committee (reference 18/LO/0025; Integrated Research Application System 225006). Written informed consent was provided by parents or guardians, and assent was provided by each child.

### Procedures

Confirmed and probable cases in the affected class were invited to participate in daily sampling as soon as possible after diagnosis and up to day 8 of treatment, followed by samples collected at weekly intervals for a further 3 weeks. These samples comprised throat swabs, hand swabs, and Columbia Blood Agar cough plates (Oxoid, Basingstoke, UK; appendix p 19). Children with scarlet fever were visited at home during the initial periods of school exclusion.

All household and classroom contacts of each case were invited to participate in weekly throat swab sampling for 4 weeks, or for a total of four occasions if the weekly cycle was interrupted by holidays. In year 2, classroom contacts were additionally asked to provide hand swabs and cough plates. To compare prevalence of carriage during an outbreak with prevalence after an outbreak, the protocol incorporated a break in the sampling schedule in year 2. This break involved a fourth sampling visit 2–3 months after the third visit, with flexibility to adjust this period to remain within the school term. Any child with clinical pharyngitis or scarlet fever was directed to their primary care physician for management; swab results were not reported to participants or to their physicians.

Routine advice on cleaning was issued before environmental sampling.^8^ Environmental swabs were obtained from 20 frequently touched items, including toys and equipment in each classroom, in week 1 of year 1. In year 2, to sample classroom air, Columbia blood agar settle plates were positioned in each of the two classrooms at heights of approximately 1·5–2·0 m for 2–3 h per week (appendix pp 19–20).

After overnight culture, DNA was extracted from any *S pyogenes* identified and *emm* genotyping was performed, followed by genome sequencing (appendix p 20). Genome sequences (appendix pp 9–13) were compared with *S pyogenes* sequences from an earlier survey of isolates associated with scarlet fever^9^ (appendix pp 14, 19–21); *emm1* and *emm4* lineages were assigned as reported.^5^, ^10^ Genome sequencing data are available from the European Nucleotide Archive using the reference PRJEB43915. To evaluate sensitivity of conventional culture, DNA extracted from culture-negative swabs from one setting was subject to *S pyogenes* ProS PCR (appendix p 19).

### Statistical analysis

Descriptive statistics only were used to analyse the data.

### Role of the funding source

The funders of the study had no role in study design, data collection, data analysis, data interpretation, or writing of the report.

## Results

**Figure 1:**
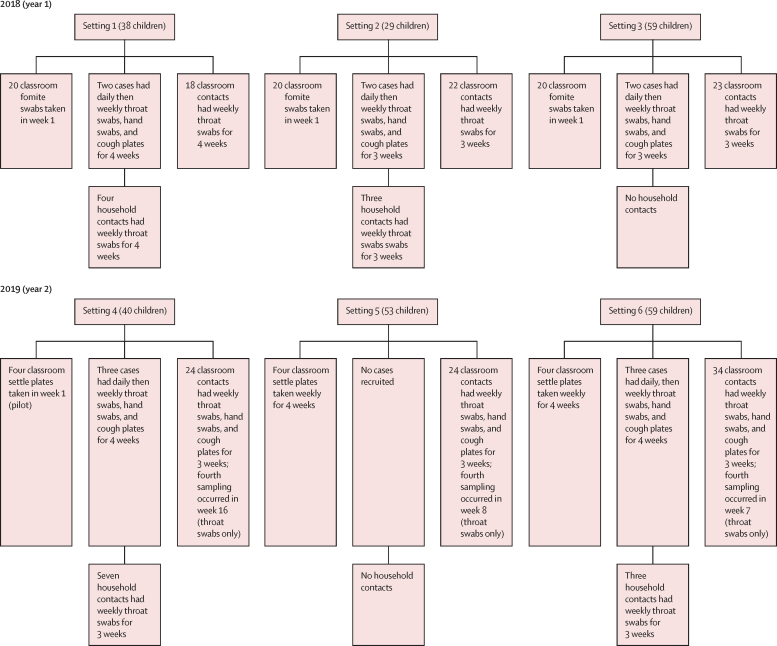
Sample types sought from cases and contacts across all six settings Two cases were identified in setting 5 but were not recruited; however, classroom contacts of these cases were recruited. The samples provided, timings, and any breaks in sampling schedule are listed in the appendix (pp 4–6).

156 outbreaks of scarlet fever in educational settings across London were reported in year 1 (2018) and 47 were reported in year 2 (2019). Six settings across five schools were included (settings 1, 2, and 3 in year 1 and settings 4, 5, and 6 in year 2), and 12 schoolchildren with scarlet fever were recruited (figure 1; appendix p 3). Two children with scarlet fever were recruited per setting in settings 1, 2, and 3 in year 1; settings 2 and 3 were different classes within the same school. Three children with scarlet fever were recruited from each of settings 4 and 6 in year 2. Although children with scarlet fever declined to participate in setting 5 in year 2, contact and environment testing was undertaken on the basis of one confirmed and one probable case. Sampling was completed by June 7, 2018, in year 1 and by June 28, 2019, in year 2. During the study, participating schools reported an increased prevalence of pharyngitis in addition to the reported cases of scarlet fever. The median attack rate for confirmed and probable cases was 5·0% (IQR 4·1–12·6) and 10·1% (7·3–24·9) with inclusion of possible cases (appendix p 3).

**Figure 2:**
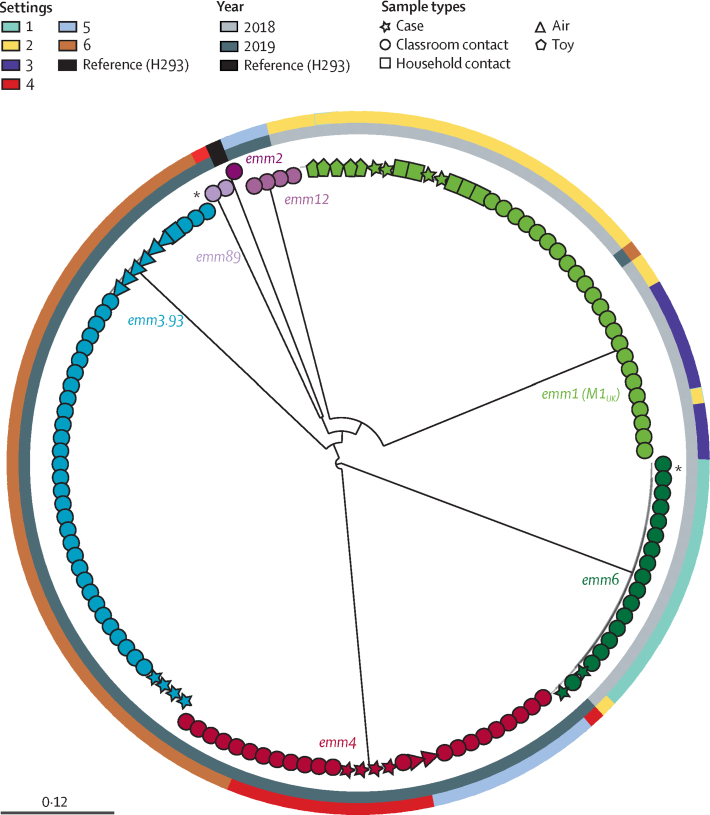
Phylogenetic tree of *Streptococcus pyogenes* isolates sequenced from cases, contacts, and the environment across all six settings Maximum likelihood phylogenetic tree constructed from 20 229 core single-nucleotide polymorphisms (without recombination regions), extracted after mapping 136 isolates of *S pyogenes* to the reference sequence H293 (*emm89*, HG316453.2). The outer rings (from outermost to innermost) represent the settings (1–6) and years of collection (2018 or 2019). The tips of the tree are coloured according to *emm* type. The shape of these tips indicates the source (ie, sample type) of individual isolates: case, classroom contact, household contact, and environmental samples from settle plate (air) or fomite (toy). In some cases and contacts, multiple isolates were detected per participant (appendix pp 4–6, 9–13). The scale bar indicates nucleotide substitution rate per site. *Singleton subtypes (*emm3.143* and *emm6.9*) within an *emm* type. A detailed analysis of each setting can be found in the appendix (pp 15–18).

Of the 12 children with scarlet fever, six children (50%) had been swabbed by primary care physicians before starting antibiotic treatment. *S pyogenes* genotypes associated with each outbreak were derived from the swabs obtained from these cases, or inferred from the *S pyogenes* isolates cultured from household contacts of cases. Genotypes were *emm6, emm1, emm4*, and *emm3.93* (figure 2). *Emm1* isolates all belonged to the newly described M1_UK_ lineage,^5^ whereas *emm4* isolates all belonged to the M4-complete lineage.^10^

11 (92%) of the 12 children with scarlet fever received antibiotic treatment prescribed by the primary care physician once a diagnosis of scarlet fever had been made. *S pyogenes* was not detected in swab samples obtained during week 1 in any of the children who had initiated antibiotic treatment before the study visit (table 1). The child who did not receive antibiotic treatment had a positive throat swab up to and including week 2, but had cleared *emm4 S pyogenes* by week 3 (table 1; appendix p 4). One contact who had a positive throat swab and cough plate on day 1 of week 1 with *emm3.93 S pyogenes* developed scarlet fever on the same day, so was also recruited as a case. Where the duration of antibiotic treatment was known in eight (67%) of the 12 cases, four children completed a 10-day course and four children took an incomplete course (including two cases prescribed a 7-day course). Of the 11 children with scarlet fever who had received antibiotic treatment, including those who completed a full 10-day course, the throat swab grew the prevailing outbreak strain of *S pyogenes* again in four (36%) children (by week 2 [one with *emm3.93*] and by week 3 [two with M1_UK_ and one with *emm3.93*]). Additionally, one of the children with the M1_UK_ strain had a positive cough plate and hand swab in week 3, despite testing negative in weeks 1 and 2 (table 1; appendix p 4).

**Table 1:** *Streptococcus pyogenes* carriage and shedding in 12 cases of scarlet fever across six settings

	**Before the study**	Week 1*			**Week 2**	**Week 3**	**Week 4**	**Week 7–8**	**Week 16**
		Day 1	Day 2	Day 3					
Antibiotic treatment†	10/12 (83%)	10/12 (83%)	11/12 (92%)	11/12 (92%)	9/12 (75%)	0/12	0/12	0/12	0/12
Positive throat swab‡	5/6 (83%)	2/12 (17%)	1/6 (17%)	1/5 (20%)	2/11 (18%)	4/10 (40%)	0/5	1/3 (33%)	1/3 (33%)
Positive cough plate	..	1/12 (8%)	0/6	0/5	0/11	1/10 (10%)	0/5	..	..
Positive hand swab	..	0/12	0/6	0/5	0/11	1/10 (10%)	0/5	..	..

Data are n/N (%). Final week of swabbing was week 4 (five cases), week 7 (three cases), or week 16 (three cases).

* Children were swabbed daily in week 1, until at least 72 h antibiotic therapy was given. Day 1 indicates first day of the study and the first swab.

† One child was identified in a contact at the start of the study, so initiated antibiotics after their first swab. One child did not receive antibiotics.

‡ Only six children were swabbed by a primary care physician before the study.

17 household contacts were enrolled into the study, representing nine households (figure 1). Of these contacts, three (18%) individuals had throat swabs that yielded *S pyogenes*, two of whom had symptomatic pharyngitis with the M1_UK_ strain and showed carriage spanning 2 or 3 weeks (appendix p 5). Indeed, the M1_UK_ strain was responsible for infection in two (67%) of three household contacts (appendix p 5).

**Figure 3:**
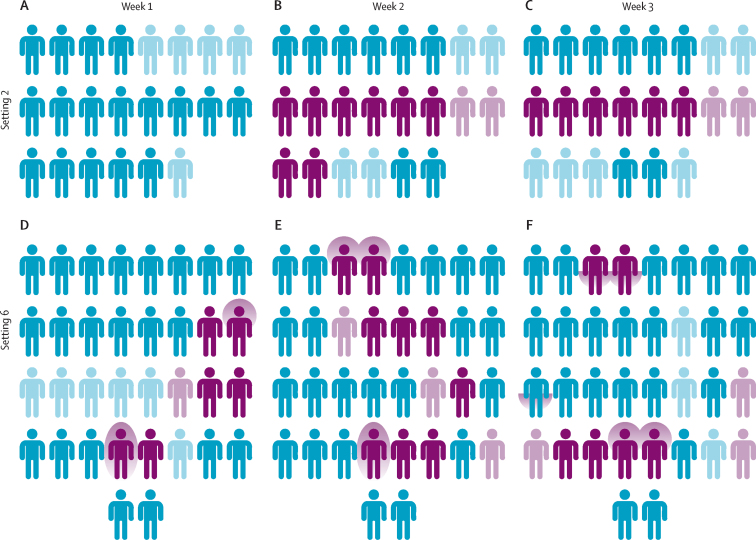
Acquisition of *emm1* (M1_UK_) and *emm3.93* strains of *Streptococcus pyogenes* by classroom contacts in settings 2 (year 1) and 6 (year 2) Each icon represents an individual child, grouped by week of swabbing, in week 1 (A), week 2 (B), and week 3 (C) in setting 2 and in week 1 (D), week 2 (E), and week 3 (F) in setting 6. The colour of each icon indicates the throat swab result: negative (blue), outbreak *S pyogenes* strain (dark purple), non-outbreak strain or genotype not confirmed (pale purple), or participant not swabbed that week (light blue). A purple glow around the icon's head indicates a positive cough plate, around the icon's hands indicates positive hand swab, and around the whole icon indicates both a positive cough plate and hand swab. Classroom contacts had throat swabs taken on a weekly basis for at least 3 weeks after a case of scarlet fever was identified.

Among all classroom contacts swabbed in schools, asymptomatic throat carriage of the outbreak *S pyogenes* strains identified by genome sequencing increased from 11 (10%) of 115 in week 1, to 34 (27%) of 126 in week 2, and 26 (24%) of 108 in week 3, before decreasing again from week 4 to weeks 7–8 (table 2). None of the 18 child contacts swabbed in week 16 were carrying *S pyogenes* (table 2). Compared with carriage of the outbreak strains, median carriage of non-outbreak *S pyogenes* was 2·8% (IQR 0·0–6·6) during the study. Transmission of the outbreak strain appeared to be particularly intense in setting 2 in year 1, where eight (44%) of 18 children swabbed were infected with the outbreak M1_UK_ strain in week 2, in addition to two household contacts, and six (38%) children were infected with the outbreak strain in week 3 (figure 3B; appendix p 6). Overall, across all six settings, more than half of eligible children participated in the study (median participation rate 52·5%, IQR 43·7–65·8; appendix p 6).

**Table 2:** Prevalence of *Streptococcus pyogenes* from throat swabs of classroom contacts across all six settings

	**Week 1**	**Week 2**	**Week 3**	**Week 4**	**Week 7–8**	**Week 16**
**Setting 1 (*emm6*)**						
Number of swabs taken	16	13	15	15	..	..
Positive for *S pyogenes*	3 (19%)	4 (31%)	7 (47%)	3 (20%)	..	..
Positive for outbreak strain	3 (19%)	4 (31%)	7 (47%)	3 (20%)	..	..
**Setting 2 (*emm1* [M1_UK_])**						
Number of swabs taken	17	18	16	Holiday	..	..
Positive for *S pyogenes*	0	10 (56%)	8 (50%)	Holiday	..	..
Positive for outbreak strain	0	8 (44%)	6 (38%)	Holiday	..	..
**Setting 3 (*emm1* [M1_UK_])**						
Number of swabs taken	19	19	Holiday	20	..	..
Positive for *S pyogenes*	2 (11%)	6 (32%)	Holiday	2 (10%)	..	..
Positive for outbreak strain	2 (11%)	6 (32%)	Holiday	2 (10%)	..	..
**Setting 4 (*emm4*)**						
Number of swabs taken	18	20	24	..	..	18
Positive for *S pyogenes*	1 (6%)	4 (20%)	4 (17%)	..	..	0
Positive for outbreak strain	0	4 (20%)	4 (17%)	..	..	0
**Setting 5 (*emm4*)**						
Number of swabs taken	17	22	22	..	23	..
Positive for *S pyogenes*	0	3 (14%)	4 (18%)	..	5 (22%)	..
Positive for outbreak strain	0	3 (14%)	3 (14%)	..	3 (13%)	..
**Setting 6 (*emm3.93*)**						
Number of swabs taken	28	34	31	..	30	..
Positive for *S pyogenes*	7 (25%)	12 (35%)	9 (29%)	..	5 (17%)	..
Positive for outbreak strain	6 (21%)	9 (26%)	6 (19%)	..	3 (10%)	..
**Total**						
Number of swabs taken	115	126	108	35	53	18
Positive for non-outbreak or unconfirmed strains	2 (2%)	5 (4%)	6 (6%)	0	4 (8%)	0
Positive for outbreak strains	11 (10%)	34 (27%)	26 (24%)	5 (14%)	6 (11%)	0

Data are n (%). Final week of sampling in year 2 was deferred to week 7 or week 16 for settings 4, 5, and 6. Outbreak strains confirmed by whole-genome sequencing. Further results are provided in the appendix (p 6).

Whole-genome sequencing was done on all viable *S pyogenes* isolates identified. Within each setting, isolates of the same genotype were clonal, with individual strains being a median of 0 single-nucleotide polymorphisms (SNPs; range 0–5 SNPs) different from other strains in that setting. This finding is consistent with a common source of transmission. Individual strains were a median of 12 SNPs (range 4–55 SNPs) different from the closest strain of the same genotype sequenced in 2014. Core genomes were more similar than was expected (appendix pp 8, 15–18).

Environmental swabs taken in settings 1, 2, and 3 in week 1 of year 1 yielded mixed bacterial growth (approximately 1 × 10^2^–1 × 10^4^ colony forming units per swab; appendix p 7). *S pyogenes* was identified from just one toy (train track) from setting 2: five colonies per swab of the outbreak strain M1_UK_ were 1–3 SNPs different to isolates obtained from children in the same class (appendix p 16).

To identify the source of ongoing transmission in the classrooms, the protocol was amended in year 2 to include hand swabs and cough plates from classroom contacts in addition to throat swabs, while settle plates were used to sample air within the classroom. In setting 4, where *emm4* predominated, the cough plate for one of four children carrying *S pyogenes* was positive in week 2, but not in subsequent weeks (appendix p 6). All isolates in setting 4 were 0 SNPs apart (appendix p 17). Cough plates were negative in setting 5, where *emm4* also predominated (and isolates were 0 SNPs apart; appendix pp 6, 17). Overall one (7%) of 14 throat swabs that were positive for *emm4* was associated with a positive cough plate. In setting 6, where the outbreak *emm3.93* strain predominated, cough plates were positive for *emm3.93* in a third of children with *emm3.*93-positive throat swabs (two of six children in week 1, three of nine in week 2, and two of six in week 3; figure 3; appendix p 6). Most strains were 0 SNPs different from one another, except for three strains that were 1, 2, and 5 SNPs apart (appendix p 18). SNPs differentiating strains within each outbreak are listed in the appendix (p 8).

In setting 4, where the outbreak *emm4* strain was identified in none of 18 classroom contacts who had a swab taken in week 1, four (20%) of 20 contacts in week 2, and four (17%) of 24 contacts in week 3, final samples from the 18 classroom contacts were taken in week 16 after an intentional break in sampling and all were negative. It was only possible to incorporate a 4-week gap for settings 5 and 6 for logistic reasons related to the timing of the school term. In setting 5, carriage of *emm4* remained steady throughout the study, identified in none of 17 classroom contacts who had a swab taken in week 1, three (14%) of 22 contacts in week 2, three (14%) of 22 contacts in week 3, and three (13%) of 23 contacts in week 7. Notably, two of three contacts with *emm4* who were positive in week 7 had also been positive in week 3. In setting 6, where *emm3.93* was identified in the throat swabs of six (21%) of 28 asymptomatic classroom contacts in week 1, in nine (26%) of 34 contacts in week 2, and in six (20%) of 30 contacts in week 3 (figure 3), carriage of *emm3.93* fell to three (10%) of 30 contacts by week 8. Notably, in this outbreak, throat swabs from two children with scarlet fever and a single household contact were positive for *emm3.93* in week 3 (appendix pp 4–5). To investigate the likelihood that culture-based sampling could be insensitive, DNA extracted from culture-negative swabs from setting 6 was subject to *S pyogenes* ProS PCR. 50 (77%) of 65 culture-negative swabs were deemed to be negative by quantitative PCR.

In year 2, four settle plates were used per classroom in settings 5 and 6 to sample room air, sited above child head height. In setting 5, one of four settle plates (placed above a whiteboard) was positive for the *emm4* strain in weeks 2 and 3 of the study (table 3). In setting 6, two of four settle plates were positive for *emm3.93* in weeks 1, 2, and 3 of the study, including plates on top of 2 m cupboards (table 3). In both settings, the *emm4* and *emm3.93 S pyogenes* strains on settle plates were identical to strains identified in children (appendix pp 17–18).

**Table 3:** Air settle plate results from settings 5 and 6 in year 2

	**Height, m**	**Week 1**	**Week 2**	**Week 3**	**Week 7–8**
**Setting 5 (*emm4*)**					
Shelf	1·5	Negative	Negative	Negative	Negative
Shelf	1·5	Negative	Negative	Negative	Negative
Shelf	1·5	Negative	Negative	Negative	Negative
Top of whiteboard	1·5	Negative	Positive	Positive	Negative
**Setting 6 (*emm3.93*)**					
Top of cupboard	2·0	Positive	Positive	Positive	Negative
Shelf	2·0	Negative	Negative	Negative	Negative
Shelf	2·0	Positive	Negative	Negative	Negative
Top of cupboard	2·0	Negative	Positive	Positive	Negative

## Discussion

This prospective, longitudinal, multicohort study of *S pyogenes* transmission was done in response to an unprecedented rise in scarlet fever notifications in England (UK). Using genome sequencing to confirm common sources of transmission, we found a high prevalence of the outbreak strain among asymptomatic classroom contacts, peaking in week 2 of our investigations. Despite antibiotic treatment and isolation of index cases for 24 h after initiation of antibiotic treatment, as well as implementation of standard hygiene measures within the classrooms, transmission within the class was observed. Enhanced sampling in year 2 showed evidence of prominent *S pyogenes* shedding by some children, and airborne dispersal of genomically identical strains in the classroom.

In the six settings investigated, *emm1* (M1_UK_) strains were involved in two outbreaks, *emm4* in two outbreaks, and *emm3.93* and *emm6* strains in one outbreak each. New lineages causing scarlet fever have been associated with national upsurges in invasive *S pyogenes,*^5^, ^11^ exemplified by emergence of M1_UK_, a lineage that expresses increased amounts of the scarlet fever toxin, speA.^5^ However, there are international differences in approach to treatment of streptococcal pharyngitis,^12^ and transmission risks are not widely considered.^13^ In addition to the public health impact of outbreaks,^2^ high attack rates in schools,^14^ and increased risk of invasive infections,^4^ there is a rationale to limit the spread of *S pyogenes* in the population, particularly where lineages such as *emm1* and *emm3*, which are independently associated with high case fatality or severe manifestations,^15^ are involved.

Current public health guidance on management of scarlet fever focuses on treatment, exclusion, and hygiene interventions, with escalation to include daily cleaning of surfaces.^8^ Although contamination of fomites, including toys, is no doubt important, we found that transmission was ongoing despite such cleaning and in the absence of frequent surface contamination with *S pyogenes*. We observed that the main source of *S pyogenes* was the children themselves. Other than foodborne outbreaks of scarlet fever,^16^ there are remarkably few recent investigations that examine transmission routes. Four contemporary studies reported outbreaks of scarlet fever with high attack rates (23–72%) in educational settings that were not controlled by standard interventions;^17^, ^18^, ^19^, ^20^ however, transmission routes were investigated in just one of these studies.^17^ Three studies described use of antibiotic treatment to end the outbreaks, treating all asymptomatic children found to be infected.^17^, ^18^, ^19^ Nevertheless, microbiological detection of *S pyogenes* to allow treatment of children found to be infected would be challenging to undertake routinely for all outbreaks of scarlet fever. Our study underlines a need for research to evaluate the potential role of molecular point-of-care tests for rapid detection of colonisation and outbreak management.

By week 3, prevalence of the genomically confirmed outbreak strain was 14–47% across all six settings, whereas carriage of non-outbreak strains was infrequent. The carriage rates detected in our study are remarkably similar to data from outbreaks with higher attack rates,^17^, ^18^, ^19^, ^20^ and to findings from studies from the pre-antibiotic era, in which 26–33% of classroom contacts with streptococcal pharyngitis were observed to carry the outbreak serotype.^21^ Our study emphasises the importance of context when measuring the carriage rates of *S pyogenes* because, outside of the springtime outbreak season in England, UK, asymptomatic carriage is estimated to be less than 6% in healthy children^22^ and less than 1% in healthy adults.^23^ Therefore, studies that report average annual rates of asymptomatic carriage do not recognise the impact of seasonal variation and outbreaks, and might provide misleading contextual information when providing recommendations.

We frequently recovered the outbreak strain among classroom contacts in week 1 of sampling, indicating that transmission had already occurred in the classroom, either from one of the index cases of scarlet fever or from another unknown source. It is possible that treatment could have been delayed in some cases. A study, also conducted in 2018, identified that a fifth of scarlet fever cases surveyed across London, UK, were initially diagnosed as a viral infection, potentially allowing transmission to continue if the affected child remained in school or was left untreated.^24^ Whether or not this situation explains outbreaks of scarlet fever merits further analysis and modelling.

It is generally believed that asymptomatic carriage is unlikely to lead to transmission, although the term carrier is more often used to refer to an individual who has been treated for *S pyogenes* pharyngitis but shows microbiological failure.^25^ In our study, the systematic increase in prevalence of outbreak strains between week 1 and week 2 among classroom contacts pointed to ongoing transmission from asymptomatic carriers, even though index cases were excluded and treated. After returning to school, four children who had scarlet fever reacquired the outbreak strains M1_UK_ (two cases) and *emm3.93* (two cases) by week 3, presumably through contact with other children with infection or through classroom air; however, these children did not develop symptomatic illness. The use of cough plates and hand swabs in our investigations showed that at least a third of asymptomatic *emm3.93* carriers were shedding more *S pyogenes* than were other children. These findings raise a question about the definition of colonisation and infection in young children, who might not be able to identify their symptoms or might be infectious without symptoms. Our study substantiates the existence of so-called heavy shedders among individuals who are apparently asymptomatic at the time of sampling, and resonates with older reports of heavy nasal shedding by nasal carriers of *S pyogenes*.^26^

Settle plates, placed in elevated locations for just 2–3 h, provided evidence of *S pyogenes* dispersal in classroom air in settings 5 and 6. The timing of settle plate positivity coincided with more intense periods of asymptomatic shedding. Settle plates provide an easy read-out in an outbreak setting and could be used to indicate a need for improved ventilation, physical distancing, or surveillance for heavy shedders. Airborne spread of streptococci causing scarlet fever was previously recognised as a threat in hospitals in the 1930s,^27^ and in military barracks in the 1940s.^28^ Contemporary outbreaks of *S pyogenes*, which are not explained by direct contact alone, suggest a need to consider not only indirect contact but also airborne transmission when developing guidelines.

The outbreak setting of scarlet fever has provided an unexpected model for understanding transmission of and immunity to *S pyogenes*. Children of the same age were exposed to a presumed similar inoculum of *S pyogenes* in class, yet only around 5% developed scarlet fever, some of whom reacquired the outbreak strain without illness by week 3, whereas others anecdotally developed pharyngitis. Other children were found to asymptomatically shed *S pyogenes* for several weeks, whereas others showed either transient colonisation or no infection at all. Our understanding of immunity to *S pyogenes* is heavily dominated by factors that influence susceptibility to invasive disease. The findings highlight a gap in our knowledge regarding mucosal immunity to *S pyogenes*, specifically whether full immunity requires antibodies to prevent streptococcal adherence, promote bacterial clearance, and inhibit streptococcal virulence factors during pharyngitis. This knowledge is potentially crucial for vaccine development. Coupled with differences in genetic susceptibility and oral microbiota, differing layers of immunity could explain to an extent why children express a range of disease phenotypes.

There are limitations to our study. Sampling of fomites was limited to single timepoints in year 1, while settle plates to sample air were only used in year 2, restricting our ability to establish the relative importance of each transmission route. Our study was based in London, UK; therefore, these findings might not be relevant to rural areas, low-income and middle-income settings, or regions with different climates. Furthermore, intensity of transmission might be seasonal. We attempted to sample outside of the main scarlet fever season, but schools were unwilling to participate; engagement was highest when anxiety about scarlet fever was greatest.

The study has shown that heavy asymptomatic shedding by a proportion of children might account for persistence of *S pyogenes* outbreaks in classroom settings. The notion of so-called super shedders and super spreaders is increasingly recognised as a source of heterogeneity in infectious disease modelling,^29^, ^30^ and is consistent with the explosive nature of some streptococcal outbreaks. Relevance to other respiratory infections is unclear. Our findings might explain the periodic failure of interventions focused on hygiene alone to curtail outbreaks of *S pyogenes*, both in the classroom and other institutional settings. The recognition of heavy asymptomatic shedding highlights a potential role for physical distancing, improved respiratory hygiene, and increased ventilation in reducing transmission of *S pyogenes* during outbreaks. As an unforeseen consequence of the COVID-19 pandemic, implementation of these measures has proven to be highly successful in halting England's scarlet fever upsurge (at least in 2020),^31^ highlighting a need for better understanding of transmission routes in preventing future upsurges.

## Data sharing

All data are included in the appendix (pp 1–21) or are available from the European Nucleotide Archive using the reference PRJEB43915.

## Declaration of interests

We declare no competing interests.

## References

[1] Guy R, Williams C, Irvine N (2014). Increase in scarlet fever notifications in the United Kingdom, 2013/2014. Euro Surveill.

[2] Lamagni T, Guy R, Chand M (2018). Resurgence of scarlet fever in England, 2014-16: a population-based surveillance study. Lancet Infect Dis.

[3] Public Health England Group A streptococcal infections: first report of seasonal activity, 2018/19. https://assets.publishing.service.gov.uk/government/uploads/system/uploads/attachment_data/file/782182/hpr0819_sf-gas.pdf.

[4] Watts V, Balasegaram S, Brown CS (2019). Increased risk for invasive group A *Streptococcus* disease for household contacts of scarlet fever cases, England, 2011-2016. Emerg Infect Dis.

[5] Lynskey NN, Jauneikaite E, Li HK (2019). Emergence of dominant toxigenic M1T1 *Streptococcus pyogenes* clone during increased scarlet fever activity in England: a population-based molecular epidemiological study. Lancet Infect Dis.

[6] Wagenvoort JH, Penders RJ, Davies BI, Lütticken R (2005). Similar environmental survival patterns of *Streptococcus pyogenes* strains of different epidemiologic backgrounds and clinical severity. Eur J Clin Microbiol Infect Dis.

[7] Kramer A, Schwebke I, Kampf G (2006). How long do nosocomial pathogens persist on inanimate surfaces? A systematic review. BMC Infect Dis.

[8] Public Health England (October 2017). Guidelines for the public health management of scarlet fever outbreaks in schools, nurseries and other childcare settings. https://assets.publishing.service.gov.uk/government/uploads/system/uploads/attachment_data/file/771139/Guidelines_for_the_public_health_management_of_scarlet_fever_outbreaks__.pdf.

[9] Chalker V, Jironkin A, Coelho J (2017). Genome analysis following a national increase in Scarlet Fever in England 2014. BMC Genomics.

[10] Remmington A, Haywood S, Edgar J, Green LR, de Silva T, Turner CE (2021). Cryptic prophages within a *Streptococcus pyogenes* genotype *emm4* lineage. Microb Genom.

[11] Al-Shahib A, Underwood A, Afshar B (2016). Emergence of a novel lineage containing a prophage in *emm*/M3 group A *Streptococcus* associated with upsurge in invasive disease in the UK. Microb Genom.

[12] Matthys J, De Meyere M, van Driel ML, De Sutter A (2007). Differences among international pharyngitis guidelines: not just academic. Ann Fam Med.

[13] National Institute for Clinical Excellence (July 23, 2008). Respiratory tract infections (self-limiting): prescribing antibiotics. https://www.nice.org.uk/guidance/cg69/evidence/full-guideline-pdf-196853293.

[14] Ryu S, Chun BC (2018). Investigation of scarlet fever outbreak in a kindergarten. Infect Chemother.

[15] Nelson GE, Pondo T, Toews KA (2016). Epidemiology of invasive group A streptococcal infections in the United States, 2005–2012. Clin Infect Dis.

[16] Yang SG, Dong HJ, Li FR (2007). Report and analysis of a scarlet fever outbreak among adults through food-borne transmission in China. J Infect.

[17] Falck G, Kjellander J (1992). Outbreak of group A streptococcal infection in a day-care center. Pediatr Infect Dis J.

[18] Feeney KT, Dowse GK, Keil AD, Mackaay C, McLellan D (2005). Epidemiological features and control of an outbreak of scarlet fever in a Perth primary school. Commun Dis Intell Q Rep.

[19] Hoebe CJ, Wagenvoort JH, Schellekens JF (2000). An outbreak of scarlet fever, impetigo and pharyngitis caused by the same *Streptococcus pyogenes* type T4M4 in a primary school. Ned Tijdschr Geneeskd.

[20] Lamden KH (2011). An outbreak of scarlet fever in a primary school. Arch Dis Child.

[21] Glover JA, Griffith F (1931). Acute tonsillitis and some of its sequels: epidemiological and bacteriological observations. BMJ.

[22] Spitzer J, Hennessy E, Neville L (2001). High group A streptococcal carriage in the Orthodox Jewish community of north Hackney. Br J Gen Pract.

[23] Pearson M, Fallowfield JL, Davey T (2017). Asymptomatic group A streptococcal throat carriage in Royal Marines recruits and young officers. J Infect.

[24] Herdman MT, Cordery R, Karo B (2021). Clinical management and impact of scarlet fever in the modern era: findings from a cross-sectional study of cases in London, 2018-2019. BMJ Open.

[25] DeMuri GP, Wald ER (2014). The group A streptococcal carrier state reviewed: still an enigma. J Pediatric Infect Dis Soc.

[26] Hamburger M, Green MJ (1946). The problem of the dangerous carrier of hemolytic streptococci; observations upon the role of the hands, of blowing the nose, of sneezing, and of coughing in the dispersal of these microorganisms. J Infect Dis.

[27] Brown WA, Allison VD (1937). Infection of the air of scarlet-fever wards with *Streptococcus pyogenes*. J Hyg (Lond).

[28] Loosli CG, Lemon HM, Wise H, Robertson OH (1948). Studies on the transmission and control of respiratory disease within army barracks: I. Hemolytic streptococcal environmental reservoirs and their relation to the nose and throat flora of military personnel. J Inf Disp.

[29] Woolhouse M (2017). Quantifying transmission. Microbiol Spectr.

[30] Adam DC, Wu P, Wong JY (2020). Clustering and superspreading potential of SARS-CoV-2 infections in Hong Kong. Nat Med.

[31] Public Health England (2019/20). Group A streptococcal infections: third report on seasonal activity in England. https://assets.publishing.service.gov.uk/government/uploads/system/uploads/attachment_data/file/887272/hpr1020_GAS-SF.pdf.

