# Supplementary material for: Frequency of transmission, asymptomatic shedding, and airborne spread of Streptococcus pyogenes in schoolchildren exposed to scarlet fever: a prospective, longitudinal, multicohort, molecular epidemiological, contact-tracing study in England, UK
Source: Lancet Microbe. 2022 May;3(5):e366–75. doi: 10.1016/S2666-5247(21)00332-3 (PMC9042792; doi:10.1016/S2666-5247(21)00332-3)
Supplement: Supplementary appendix [file mmc1.pdf]

THE LANCET
Microbe

Supplementary appendix

This appendix formed part of the original submission and has been peer reviewed. We post it as supplied by the authors.

Supplement to: Cordery R, Purba AK, Begum L, et al. Frequency of transmission, asymptomatic shedding, and airborne spread of *Streptococcus pyogenes* in schoolchildren exposed to scarlet fever: a prospective, longitudinal, multicohort, molecular epidemiological, contact-tracing study in England, UK. *Lancet Microbe* 2022; published online March 10. https://doi.org/10.1016/S2666-5247(21)00332-3.

Supplementary Appendix for Cordery et al

Contents

		Page
Supplementary Table 1.	Case Definitions	2
Supplementary Table 2.	Study Site Recruitment	3
Supplementary Table 3.	Cases in each setting- detailed results	4
Supplementary Table 4.	Household contacts in each setting- detailed results	5
Supplementary Table 5.	School contacts in each setting- detailed results	6
Supplementary Table 6.	Surface swab results (settings 1-3, 2018)	7
Supplementary Table 7.	SNPs between isolates within each cluster	8
Supplementary Table 8.	Accession numbers of genomes from each setting	9-13
Supplementary Table 9.	Genomes from Chalker et al (ref.12) used for analysis	14
Supplementary Figure S1.	Phylogenetic relationship of *emm*6 strains (setting 1).	15
Supplementary Figure S2.	Phylogenetic relationship of *emm*1 strains (settings 2 & 3).	16
Supplementary Figure S3.	Phylogenetic relationship of *emm*4 strains (settings 4 & 5).	17
Supplementary Figure S4.	Phylogenetic relationship of *emm*3 strains (setting 6).	18
Supplementary Methods		19-21
Suppl. References		21

Supplementary Table 1. Scarlet Fever Case Definitions

**Definitions**	
Confirmed	Clinical diagnosis of Scarlet Fever by a health professional and *S. pyogenes* detected on a throat swab
Probable	Clinical diagnosis of Scarlet Fever by a health professional
Possible	Case reported by a reliable source (e.g. nursery manager, school secretary), presenting with signs and symptoms consistent with scarlet fever, and a close epidemiological link e.g. household contact of a confirmed case; or attending school where there is a confirmed scarlet fever outbreak<br>Cases reported by a health professional where scarlet fever is part of a differential diagnosis and other infections may be just as likely.
Carriage	Throat swab positive for *S. pyogenes* in asymptomatic individual
Outbreak	2 or more probable or confirmed cases of scarlet fever with epidemiological link in time, place and person i.e. in children or staff member, within 10 days of each other in the same school or nursery class.

Inclusion criteria for participating schools were two confirmed or probable scarlet fever cases aged 2-8 years from the same class within ten days of each other, with the most recent case arising in the preceding 48h. Cases were prospectively swabbed if they were confirmed or probable.

Supplementary Table 2. Study Site case and household recruitment by setting

	Age group (years)	Inferred outbreak emm type	Final scarlet fever outbreak size	Cases recruited	Whole class size	Attack rate in setting*	Household contacts recruited
1	3-4	Emm6	2 confirmed	2	38	5.2%	4
2	5-6	Emm1 ($M1_{UK}$)	4 probable<br>9 possible	2	29	4.5-14.8%	3
3	4-5	Emm1 ($M1_{UK}$)		2	59		0
4	3-4	Emm4	4 confirmed<br>4 probable<br>6 possible	3	40	20-35%	7
5	4-5	Emm4	1 confirmed<br>1 probable<br>3 possible	0	53	3.8-9.4%	0
6	4-5	Emm3.93	3 probable<br>3 possible	3	59	5.0-10.1%	3

*Attack rate range derived from (confirmed + probable) and (confirmed + probable +possible) cases.

Supplementary Table 3. Cases in each setting- detailed results

		S. pyogenes swab results, no. of children (outbreak strain confirmed by WGS)								
	GP swab Y/N (result)	Week 1				Week 2	Week 3	Week 4	Week 7/8	Week 16
		Day 1	Day 2	Day 3	Day 4					
Setting 1										
**Case 1**	Y (**+**)									
Throat		NEG	NEG	NEG	NEG	NEG	NEG	NEG		
Cough		NEG	NEG	NEG	NEG	NEG	NEG	NEG		
Hand		NEG	NEG	NEG	NEG	NEG	NEG	NEG		
**Case 2**	Y (**+**)									
Throat		NEG	NEG	NEG	NEG	NEG	NEG	NEG		
Cough		NEG	NEG	NEG	NEG	NEG	NEG	NEG		
Hand		NEG	NEG	NEG	NEG	NEG	NEG	NEG		
Setting 2										
**Case 1**	N									
Throat		NEG	NEG	ND	ND	NEG	**+**	ND		
Cough		NEG	NEG	ND	ND	NEG	**+**	ND		
Hand		NEG	NEG	ND	ND	NEG	**+**	ND		
**Case 2**	N									
Throat		NEG	NEG	NEG	ND	NEG	**+**	ND		
Cough		NEG	NEG	NEG	ND	NEG	NEG	ND		
Hand		NEG	NEG	NEG	ND	NEG	NEG	ND		
Setting 3										
**Case 1**	N									
Throat		NEG	ND	ND	ND	NEG	ND	NEG		
Cough		NEG	ND	ND	ND	NEG	ND	NEG		
Hand		NEG	ND	ND	ND	NEG	ND	NEG		
**Case 2**	N									
Throat		NEG	ND	ND	ND	ND	ND	NEG		
Cough		NEG	ND	ND	ND	ND	ND	NEG		
Hand		NEG	ND	ND	ND	ND	ND	ND		
Setting 4										
**Case 1**	Y (-)									
Throat		**+**	**+**	**+**	ND	**+**	NEG			**+**
Cough		NEG	NEG	NEG	ND	NEG	NEG			ND
Hand		NEG	NEG	NEG	ND	NEG	NEG			ND
**Case 2**	Y (**+**)									
Throat		NEG	NEG	NEG	ND	NEG	NEG			NEG
Cough		NEG	NEG	NEG	ND	NEG	NEG			ND
Hand		NEG	NEG	NEG	ND	NEG	NEG			ND
**Case 3***	Y (**+**)									
Throat		ND	ND	ND	ND	NEG	NEG	NEG		NEG
Cough		ND	ND	ND	ND	NEG	NEG	NEG		ND
Hand		ND	ND	ND	ND	NEG	NEG	NEG		ND
Setting 6										
**Case 1**	N									
Throat		NEG	ND	ND	ND	**+**	**+**		**+**	
Cough		NEG	ND	ND	ND	NEG	NEG		ND	
Hand		NEG	ND	ND	ND	NEG	NEG		ND	
**Case 2**	Y (**+**)									
Throat		NEG	ND	ND	ND	NEG	**+**		NEG	
Cough		NEG	ND	ND	ND	NEG	NEG		ND	
Hand		NEG	ND	ND	ND	NEG	NEG		ND	
**Case 3****	N									
Throat		**+**	ND	ND	ND	NEG	NEG		NEG	
Cough		**+**	ND	ND	ND	NEG	NEG		ND	
Hand		NEG	ND	ND	ND	NEG	NEG		ND	

Positive *S. pyogenes* result indicated by '+'; negative by 'NEG'. Hatched cells indicate intentional pause in study
*Case 3 in setting 4 was studied weekly only; **Case 3 in setting 6 was identified on day 1 of the study (initially recruited as a contact). Abbreviations: Hol, school holiday (unable to sample); WGS, whole genome sequencing; GP, General practitioner (primary care physician); Y, yes; N, no; ND, not done. Setting 5, no cases recruited.

Supplementary Table 4. Household contacts in each setting- detailed results

			Week 1	Week 2	Week 3	Week 4
Setting 1 emm6						
Case1	HHC1		NEG	NEG	NEG	NEG
	HHC2		NEG	NEG	NEG	NEG
Case 2	HHC1		NEG	NEG	NEG	ND
	HHC2		ND	NEG	NEG	ND
Setting 2 emm1 ($M1_{UK}$)						
Case 1	HHC1		**+**	**+**	NEG	
Case 2	HHC1		NEG	NEG	NEG	
	HHC2		**+**	**+**	**+**	
Setting 4 emm4						
Case 1	HHC1		NEG	NEG	NEG	
	HHC2		NEG	NEG	NEG	
	HHC3		NEG	NEG	ND	
Case 2	HHC 1		NEG	NEG	NEG	
	HHC2		ND	ND	NEG	
Case 3	HHC1		NEG	NEG	NEG	
	HHC2		NEG	NEG	NEG	
Setting 6 emm3.93						
Case 1	HHC1		NEG	NEG	NEG	
	HHC2		NEG	NEG	**+**	
Case 2	HHC1		NEG	NEG	ND	
Overall Household contacts						
Participant total			17	17	17	4
Swabs taken			15	16	16	2
S. pyogenes positive (%)			2 (13.3)	2 (12.5)	2 (12.5)	0 (0)
Outbreak strain (%)			2 (13.3)	2 (12.5)	2 (12.5)	0 (0)

Positive *S. pyogenes* result indicated by '+'; negative by 'NEG'.
Abbreviations. HHC, household contact.

Supplementary Table 5. School contacts in each setting- detailed results

	S. pyogenes sample results, Number of children (outbreak strain confirmed by *emm* type or WGS)						
	Week 1	Week2	Week 3	Week 4	Week 7/8	Week 16	Whole class size
Setting 1							
TS Positive	3 (3)	4 (4)	7 (7)	3[Ψ] (3)			38
TS Negative	13	9	8	12			
TS swab total	16	13	15	15			
Absent/Ref	2	5	3	3			
Participant total	18	18	18	18			
Setting 2							
TS Positive	0	10 (8)[ΨΨΨ]	8 (6)	Hol			29
TS Negative	17	8	8	Hol			
TS swab total	17	18	16	-			
Absent/Ref	5	4	6	Hol			
Participant total	22	22	22	Hol			
Setting 3							
TS Positive	2 (2)	6 (6)	Hol	2 (2)			59
TS Negative	17	13	Hol	18			
TS swab total	19	19	-	20			
Absent/Ref	4	4	Hol	3			
Participant total	23	23	Hol	23			
Setting 4							
TS Positive	1 (0)	4 (4)	4 (4)			0	40
TS Negative	17	16	20			18	
TS swab total	18	20	24			18	
Cough positive	0	1 (1)	0			ND	
Hand positive	0	0	0			ND	
Absent/Ref	0	4	0			7	
Participant total	18	24	24			25	
Setting 5							
TS Positive	0	3 (3)	4 (3)		5 (3)		53
TS Negative	17	19	18		18		
TS swab total	17	22	22		23		
Cough positive	0	0	0		ND		
Hand positive	0	0	0		ND		
Absent/Ref	1	1	2		1		
Participant total	18	23	24		24		
Setting 6♣							
TS Positive	7[Ψ] (6)	12[Ψ] (9)	9[Ψ] (6)		5[ΨΨ] (3)		59
TS Negative	21	22	22		25		
TS swab total	28	34	31		30		
Cough positive	2 (2)	3 (3)	2 (2)		ND		
Hand positive	1 (1)	1 (1)	3[Ψ] (2)		ND		
Absent/Ref	2	0	4		4		
Participant total	30	34	34		34		

Hatched cells indicate intentional pause in study (Settings 4-6, a break between 3rd week and final week was incorporated.)
Hol, unable to sample as school holiday; TS, throat swab; ND, not done;
♣Setting 6; one contact in week 1 with positive throat swab and cough plate was subsequently diagnosed as a case and given antibiotics, with all samples subsequently negative and is shown for completeness; week 3 samples taken at end of week 2 due to school holiday.
[Ψ]one, [ΨΨ]two, or [ΨΨΨ]three samples have no WGS.

Supplementary Table 6. Surface swab results (settings 1-3, year 1)

Setting	Sample type	Colony count	Setting	Sample type	Colony count	Setting	Sample type	Colony count
1	Construction toy	100	2	Activity table	350	3	Snack table	10,000
	Construction toy	200		Activity table	450		Wooden stool	1,980
	Construction toy	100		Wooden track*	1000		Smartboard screen	60
	Construction toy	100		Magnetic letter	150		Book	100
	Construction toy	150		Magnetic letter	<25		Magnifier handle	340
	Toy car	300		Lego brick	100		Ipad screen	720
	Toy car	250		Smart screen	100		Door handle	60
	Book	400		Book	200		Small bin	400
	Book	200		Book	500		Lego table	320
	Large toy car	350		iPad	950		Activity table	1,600
	Play surface	100		iPad	1000		Dry wipe pen	<20
	Toy animal	200		Chair	200		Activity table	100
	Toy animal	350		Jigsaw piece	100		Paint table	180
	Book	500		Toy truck	250		Glue bottle	420
	Book	400		Plastic scissors	550		Book	220
	Construction parts	180		Plastic animal	200		Toy Screwdriver	180
	Construction parts	200		Book	100		Plastic apron	460
	Toy cooker	320		Phonics game	3000		Paint brush handle	1,320
	Toy shell	340		Counting toy	200		Toy animal	400
	Plastic money	200		Glue stick	200		Chair	380

Colony counts reflect mixed flora. No beta haemolytic streptococci except sample ‘*’ yielding 5 colonies of *S. pyogenes*

Supplementary Table 7. SNPs between isolates within each cluster

Classes	Emm type	PosGene	ID	Gene Name	Gene Product	SNP	Residue	Type of mutation	Variant prediction
**Setting 1**	Emm6	155402	M6_RS00980	NA	deoxynucleoside kinase	135T>C	Asp45Asp	Syn	Low
		910654	M6_RS04595	*guaA*	glutamine-hydrolyzing GMP synthase	201C>T	Tyr67Tyr	Syn	Low
		1277861	M6_RS06460	*arcC*	carbamate kinase	279T>C	Asn93Asn	Syn	Low
		1323939	M6_RS06670	NA	Glycoside hydrolase family 125 protein	180C>T	Ser60Ser	Syn	Low
		1880406	M6_RS09345	*hasB*	UDP-glucose 6-dehydrogenase HasB	172C>T	Gln58*	Stop	High
		1881063	M6_RS09345	*hasB*	UDP-glucose 6-dehydrogenase HasB	829C>A	Gln277Lys	Non-Syn	Moderate
		1894721	x	x	x	x		Intragenic	x
**Setting 2**	Emm1	14547	M5005_RS00060	NA	ATP-dependent metallopeptidase FtsH/Yme1/Tma family protein	1743T>C	Arg581Arg	Syn	Low
		907441	M5005_RS04580	*guaA*	glutamine-hydrolyzing GMP synthase	1540C>T	Pro514Ser	Non-Syn	Moderate
		1011845	M5005_RS05145	*ssb*	Single-stranded DNA-binding protein	215G>T	Gly72Val	Non-Syn	Moderate
		1477185	M5005_RS07535	NA	MptD family putative ECF transporter S Component	448A>G	Thr150Ala	Non-Syn	Moderate
**Setting 3**	Emm1	43445	M5005_RS00285	*purN*	phosphoribosylglycinamide formyltransferase	48C>A	Val16Va	Syn	Low
		940218	M5005_RS04730	NA	M1 family metallopeptidase	1098A>G	Pro366Pro	Syn	Low
		1255526	x	x	x	x		intragenic	x
**Setting 4**	Emm4	None	None	None	None	None	None	None	None
**Setting 5**	Emm4	None	None	None	None	None	None	None	None
**Setting 6**	Emm3	1882546	SpyM3_1852	*hasB*	putative UDP-glucose 6-dehydrogenase	754C>T	Pro252Ser	Non-Syn	Moderate
**Setting 4 and Setting 6**	Emm4	899513	MGAS10750_Spy 0941	NA	Xaa-His dipeptidase	1268C>A	Thr423Lys	Non-Syn	Moderate
		1010790	MGAS10750_Spy 1067	NA	hypothetical protein	913G>A	Asp305Asn	Non-Syn	Moderate
		1026739	MGAS10750_Spy 1079	NA	ATPase associated with chromosome architecture/replication	121G>A	Ala41Thr	Non-Syn	Moderate
		1450398	MGAS10750_Spy 1513	NA	PTS system, galactose-specific IIB component	62C>T	Ala21Val	Non-Syn	Moderate
		1735343	x	x	x	x		Intragenic	x

Supplementary Table 8 Accession numbers of bacterial genomes from each setting

Sample ID	Year	Setting	Source	Sample type	Timepoint (1-4)	emm typing	MLST	Accession*	Unique Name
A_C1_T_001	2019	Setting 4	Case	Throat swab	1	EMM4.0	39	ERS6110953	SAMEA8426050
A_C1_T_002	2019	Setting 4	Case	Throat swab	1	EMM4.0	39	ERS6110954	SAMEA8426051
A_C1_T_003	2019	Setting 4	Case	Throat swab	1	EMM4.0	39	ERS6110955	SAMEA8426052
A_C1_T_008	2019	Setting 4	Case	Throat swab	2	EMM4.0	39	ERS6110956	SAMEA8426053
A_C1_T_106	2019	Setting 4	Case	Throat swab	4	EMM6.0	382	ERS6110957	SAMEA8426054
A_CC18_C_008	2019	Setting 4	CC	Cough plate	2	EMM4.0	39	ERS6110958	SAMEA8426055
A_CC18_T_008	2019	Setting 4	CC	Throat swab	2	EMM4.0	39	ERS6110959	SAMEA8426056
A_CC18_T_015	2019	Setting 4	CC	Throat swab	3	EMM4.0	39	ERS6110960	SAMEA8426057
A_CC22_T_008	2019	Setting 4	CC	Throat swab	2	EMM4.0	39	ERS6110961	SAMEA8426058
A_CC22_T_015	2019	Setting 4	CC	Throat swab	3	EMM4.0	39	ERS6110962	SAMEA8426059
A_CC27_T_008	2019	Setting 4	CC	Throat swab	2	EMM4.0	39	ERS6110963	SAMEA8426060
A_CC27_T_015	2019	Setting 4	CC	Throat swab	3	EMM4.0	39	ERS6110964	SAMEA8426061
A_CC28_T_008	2019	Setting 4	CC	Throat swab	2	EMM4.0	39	ERS6110965	SAMEA8426062
A_CC28_T_015	2019	Setting 4	CC	Throat swab	3	EMM4.0	39	ERS6110966	SAMEA8426063
A_CC31_T_001	2019	Setting 4	CC	Throat swab	1	EMM89.0	101	ERS6110967	SAMEA8426064
B_CC05_T_008	2019	Setting 5	CC	Throat swab	3	EMM12.0	36	ERS6110968	SAMEA8426065
B_CC05_T_046	2019	Setting 5	CC	Throat swab	4	EMM12.0	36	ERS6110969	SAMEA8426066
B_CC21_T_046	2019	Setting 5	CC	Throat swab	4	EMM2.0	55	ERS6110970	SAMEA8426067
B_CC22_T_008	2019	Setting 5	CC	Throat swab	3	EMM4.0	39	ERS6110971	SAMEA8426068
B_CC22_T_046	2019	Setting 5	CC	Throat swab	4	EMM4.0	39	ERS6110972	SAMEA8426069
B_CC34_T_001	2019	Setting 5	CC	Throat swab	2	EMM4.0	39	ERS6110973	SAMEA8426070
B_CC34_T_008	2019	Setting 5	CC	Throat swab	3	EMM4.0	39	ERS6110974	SAMEA8426071
B_CC41_T_001	2019	Setting 5	CC	Throat swab	2	EMM4.0	39	ERS6110975	SAMEA8426072
B_CC44_T_001	2019	Setting 5	CC	Throat swab	2	EMM4.0	39	ERS6110976	SAMEA8426073
B_CC44_T_008	2019	Setting 5	CC	Throat swab	3	EMM4.0	39	ERS6110977	SAMEA8426074
B_CC44_T_046	2019	Setting 5	CC	Throat swab	4	EMM4.0	39	ERS6110978	SAMEA8426075
B_CC49_T_046	2019	Setting 5	CC	Throat swab	4	EMM4.0	39	ERS6110979	SAMEA8426076
B_E4_S_001	2019	Setting 5	Air	Settle plate	2	EMM4.0	39	ERS6110980	SAMEA8426077
B_E4_S_008	2019	Setting 5	Air	Settle plate	3	EMM4.0	39	ERS6110981	SAMEA8426078

C_C1_T_010	2019	Setting 6	Case	Throat swab	3	EMM3.93	315	ERS6110982	SAMEA8426079
C_C1_T_046	2019	Setting 6	Case	Throat swab	4	EMM3.93	315	ERS6110983	SAMEA8426080
C_C2_T_010	2019	Setting 6	Case	Throat swab	3	EMM3.93	315	ERS6110984	SAMEA8426081
C_C1_T_007	2019	Setting 6	Case	Throat swab	2	EMM3.93	315	ERS6110985	SAMEA8426082
C_CC02_T_007	2019	Setting 6	CC	Throat swab	2	EMM3.93	315	ERS6110986	SAMEA8426083
C_CC07_T_007	2019	Setting 6	CC	Throat swab	2	EMM4.0	39	ERS6110987	SAMEA8426084
C_CC09_C_007	2019	Setting 6	CC	Cough plate	2	EMM3.93	315	ERS6110988	SAMEA8426085
C_CC09_H_010	2019	Setting 6	CC	Hand swab	3	EMM3.93	315	ERS6110989	SAMEA8426086
C_CC09_T_007	2019	Setting 6	CC	Throat swab	2	EMM3.93	315	ERS6110990	SAMEA8426087
C_CC13_T_007	2019	Setting 6	CC	Throat swab	2	EMM3.93	315	ERS6110991	SAMEA8426088
C_CC13_T_046	2019	Setting 6	CC	Throat swab	4	EMM3.93	315	ERS6110992	SAMEA8426089
C_CC14_C_001	2019	Setting 6	CC	Cough plate	1	EMM3.93	315	ERS6110993	SAMEA8426090
C_CC14_T_001	2019	Setting 6	CC	Throat swab	1	EMM3.93	315	ERS6110994	SAMEA8426091
C_CC15_C_007	2019	Setting 6	CC	Cough plate	2	EMM3.93	315	ERS6110995	SAMEA8426092
C_CC15_H_010	2019	Setting 6	CC	Hand swab	3	EMM3.93	315	ERS6110996	SAMEA8426093
C_CC15_T_007	2019	Setting 6	CC	Throat swab	2	EMM3.93	315	ERS6110997	SAMEA8426094
C_CC15_T_010	2019	Setting 6	CC	Throat swab	3	EMM3.93	315	ERS6110998	SAMEA8426095
C_CC15_T_046	2019	Setting 6	CC	Throat swab	4	EMM3.93	315	ERS6110999	SAMEA8426096
C_CC16_T_010	2019	Setting 6	CC	Throat swab	3	EMM3.93	315	ERS6111000	SAMEA8426097
C_CC21_T_010	2019	Setting 6	CC	Throat swab	3	EMM3.93	315	ERS6111001	SAMEA8426098
C_CC22_T_001	2019	Setting 6	CC	Throat swab	1	EMM3.93	315	ERS6111002	SAMEA8426099
C_CC22_T_007	2019	Setting 6	CC	Throat swab	2	EMM3.93	315	ERS6111003	SAMEA8426100
C_CC24_T_007	2019	Setting 6	CC	Throat swab	2	EMM3.93	315	ERS6111004	SAMEA8426101
C_CC28_T_001	2019	Setting 6	CC	Throat swab	1	EMM4.0	39	ERS6111005	SAMEA8426102
C_CC28_T_007	2019	Setting 6	CC	Throat swab	2	EMM4.0	39	ERS6111006	SAMEA8426103
C_CC34_T_010	2019	Setting 6	CC	Throat swab	3	EMM4.0	39	ERS6111007	SAMEA8426104
C_CC38_C_010	2019	Setting 6	CC	Cough plate	3	EMM3.93	315	ERS6111008	SAMEA8426105
C_CC38_T_001	2019	Setting 6	CC	Throat swab	1	EMM3.93	315	ERS6111009	SAMEA8426106
C_CC38_T_007	2019	Setting 6	CC	Throat swab	2	EMM3.93	315	ERS6111010	SAMEA8426107
C_CC38_T_010	2019	Setting 6	CC	Throat swab	3	EMM3.93	315	ERS6111011	SAMEA8426108
C_CC39_T_007	2019	Setting 6	CC	Throat swab	2	EMM3.93	315	ERS6111012	SAMEA8426109

C_CC42_C_001	2019	Setting 6	CC	Cough plate	1	EMM3.93	315	ERS6111013	SAMEA8426110
C_CC42_C_007	2019	Setting 6	CC	Cough plate	2	EMM3.93	315	ERS6111014	SAMEA8426111
C_CC42_C_010	2019	Setting 6	CC	Cough plate	3	EMM3.93	315	ERS6111015	SAMEA8426112
C_CC42_H_001	2019	Setting 6	CC	Hand swab	1	EMM3.93	315	ERS6111016	SAMEA8426113
C_CC42_H_007	2019	Setting 6	CC	Hand swab	2	EMM3.93	315	ERS6111017	SAMEA8426114
C_CC42_T_007	2019	Setting 6	CC	Throat swab	2	EMM3.93	315	ERS6111018	SAMEA8426115
C_CC42_T_010	2019	Setting 6	CC	Throat swab	3	EMM3.93	315	ERS6111019	SAMEA8426116
C_CC51_T_010	2019	Setting 6	CC	Throat swab	3	EMM1.0	28	ERS6111020	SAMEA8426117
C_CC55_T_001	2019	Setting 6	CC	Throat swab	1	EMM3.93	315	ERS6111021	SAMEA8426118
C_CC55_T_010	2019	Setting 6	CC	Throat swab	3	EMM3.143	315	ERS6111022	SAMEA8426119
C_CC58_T_001	2019	Setting 6	CC	Throat swab	1	EMM3.93	315	ERS6111023	SAMEA8426120
C_CC58_T_046	2019	Setting 6	CC	Throat swab	4	EMM3.93	315	ERS6111024	SAMEA8426121
C_E1_S_001	2019	Setting 6	Air	Settle plate	1	EMM3.93	315	ERS6111025	SAMEA8426122
C_E1_S_007	2019	Setting 6	Air	Settle plate	2	EMM3.93	315	ERS6111026	SAMEA8426123
C_E1_S_010	2019	Setting 6	Air	Settle plate	3	EMM3.93	315	ERS6111027	SAMEA8426124
C_E3_S_001	2019	Setting 6	Air	Settle plate	1	EMM3.93	315	ERS6111028	SAMEA8426125
C_E4_S_007	2019	Setting 6	Air	Settle plate	2	EMM3.93	315	ERS6111029	SAMEA8426126
C_E4_S_010	2019	Setting 6	Air	Settle plate	3	EMM3.93	315	ERS6111030	SAMEA8426127
C_H2_T_010	2019	Setting 6	HHC	Throat swab	3	EMM3.93	315	ERS6111031	SAMEA8426128
A_C1_T_W	2018	Setting 1	Case	Throat swab	0	EMM6.0	382	ERS6111032	SAMEA8426129
A_CC00_T_W1	2018	Setting 1	CC	Throat swab	1	EMM6.0	382	ERS6111033	SAMEA8426130
A_CC00_T_W3	2018	Setting 1	CC	Throat swab	3	EMM6.0	382	ERS6111034	SAMEA8426131
A_CC11_T_W1	2018	Setting 1	CC	Throat swab	1	EMM6.0	382	ERS6111035	SAMEA8426132
A_CC11_T_W2	2018	Setting 1	CC	Throat swab	2	EMM6.0	382	ERS6111036	SAMEA8426133
A_CC04_T_W2	2018	Setting 1	CC	Throat swab	2	EMM6.0	382	ERS6111037	SAMEA8426134
A_CC04_T_W3	2018	Setting 1	CC	Throat swab	3	EMM6.0	382	ERS6111038	SAMEA8426135
A_CC08_T_W3	2018	Setting 1	CC	Throat swab	3	EMM6.0	382	ERS6111039	SAMEA8426136
A_CC09_T_W3	2018	Setting 1	CC	Throat swab	3	EMM6.0	382	ERS6111040	SAMEA8426137
A_CC09_T_W4	2018	Setting 1	CC	Throat swab	4	EMM6.0	382	ERS6111041	SAMEA8426138
A_CC10_T_W1	2018	Setting 1	CC	Throat swab	1	EMM6.9	382	ERS6111042	SAMEA8426139
A_CC13_T_W2	2018	Setting 1	CC	Throat swab	2	EMM6.0	382	ERS6111043	SAMEA8426140
A_CC13_T_W3	2018	Setting 1	CC	Throat swab	3	EMM6.0	382	ERS6111044	SAMEA8426141
A_CC13_T_W4	2018	Setting 1	CC	Throat swab	4	EMM6.0	382	ERS6111045	SAMEA8426142

A_CC14_T_W3	2018	Setting 1	CC	Throat swab	3	EMM6.0	382	ERS6111046	SAMEA8426143
A_CC15_T_W2	2018	Setting 1	CC	Throat swab	2	EMM6.0	382	ERS6111047	SAMEA8426144
A_CC15_T_W3	2018	Setting 1	CC	Throat swab	3	EMM6.0	382	ERS6111048	SAMEA8426145
B_C1_C_W3	2018	Setting 2	Case	Cough plate	3	EMM1.0	28	ERS6111049	SAMEA8426146
B_C1_H_W3	2018	Setting 2	Case	Hand swab	3	EMM1.0	28	ERS6111050	SAMEA8426147
B_C1_T_W3	2018	Setting 2	Case	Throat swab	3	EMM1.0	28	ERS6111051	SAMEA8426148
B_C2_T_W3	2018	Setting 2	Case	Throat swab	3	EMM1.0	28	ERS6111052	SAMEA8426149
B_C1_HH_W1	2018	Setting 2	HHC	Household	1	EMM1.0	28	ERS6111053	SAMEA8426150
B_C2_HH_W1	2018	Setting 2	HHC	Household	1	EMM1.0	28	ERS6111054	SAMEA8426151
B_C1_HH_W2	2018	Setting 2	HHC	Household	2	EMM1.0	28	ERS6111055	SAMEA8426152
B_C2_HH_W2	2018	Setting 2	HHC	Household	2	EMM1.0	28	ERS6111056	SAMEA8426153
B_C2_HH_W3	2018	Setting 2	HHC	Household	3	EMM1.0	28	ERS6111057	SAMEA8426154
B_CC01_T_W2	2018	Setting 2	CC	Throat swab	2	EMM1.0	28	ERS6111058	SAMEA8426155
B_CC04_T_W2	2018	Setting 2	CC	Throat swab	2	EMM1.0	28	ERS6111059	SAMEA8426156
B_CC04_T_W3	2018	Setting 2	CC	Throat swab	3	EMM1.0	28	ERS6111060	SAMEA8426157
B_CC06_T_W2	2018	Setting 2	CC	Throat swab	2	EMM1.0	28	ERS6111061	SAMEA8426158
B_CC06_T_W3	2018	Setting 2	CC	Throat swab	3	EMM1.0	28	ERS6111062	SAMEA8426159
B_CC08_T_W2	2018	Setting 2	CC	Throat swab	2	EMM1.0	28	ERS6111063	SAMEA8426160
B_CC08_T_W3	2018	Setting 2	CC	Throat swab	3	EMM1.0	28	ERS6111064	SAMEA8426161
B_CC09_T_W3	2018	Setting 2	CC	Throat swab	3	EMM1.0	28	ERS6111065	SAMEA8426162
B_CC10_T_W2	2018	Setting 2	CC	Throat swab	2	EMM1.0	28	ERS6111066	SAMEA8426163
B_CC10_T_W3	2018	Setting 2	CC	Throat swab	3	EMM1.0	28	ERS6111067	SAMEA8426164
B_CC11_T_W2	2018	Setting 2	CC	Throat swab	2	EMM12.0	36	ERS6111068	SAMEA8426165
B_CC11_T_W3	2018	Setting 2	CC	Throat swab	3	EMM12.0	36	ERS6111069	SAMEA8426166
B_CC14_T_W2	2018	Setting 2	CC	Throat swab	2	EMM1.0	28	ERS6111070	SAMEA8426167
B_CC14_T_W3	2018	Setting 2	CC	Throat swab	3	EMM1.0	28	ERS6111071	SAMEA8426168
B_CC19_T_W3	2018	Setting 2	CC	Throat swab	3	EMM6.0	382	ERS6111072	SAMEA8426169
B_E1_Ty_W	2018	Setting 2	Toy	Toys	1	EMM1.0	28	ERS6111073	SAMEA8426170
B_E2_Ty_W	2018	Setting 2	Toy	Toys	1	EMM1.0	28	ERS6111074	SAMEA8426171
B_E3_Ty_W	2018	Setting 2	Toy	Toys	1	EMM1.0	28	ERS6111075	SAMEA8426172
B_E4_Ty_W	2018	Setting 2	Toy	Toys	1	EMM1.0	28	ERS6111076	SAMEA8426173
B_E5_Ty_W	2018	Setting 2	Toy	Toys	1	EMM1.0	28	ERS6111077	SAMEA8426174
BR_CC12_T_W2	2018	Setting 3	CC	Throat swab	2	EMM1.0	28	ERS6111078	SAMEA8426175

BR_CC12_T_W4	2018	Setting 3	CC	Throat swab	4	EMM1.0	28	ERS6111079	SAMEA8426176
BR_CC17_T_W1	2018	Setting 3	CC	Throat swab	1	EMM1.0	28	ERS6111080	SAMEA8426177
BR_CC17_T_W1_2	2018	Setting 3	CC	Throat swab	2	EMM1.0	28	ERS6111081	SAMEA8426178
BR_CC17_T_W2	2018	Setting 3	CC	Throat swab	2	EMM1.0	28	ERS6111082	SAMEA8426179
BR_CC18_T_W2	2018	Setting 3	CC	Throat swab	2	EMM1.0	28	ERS6111083	SAMEA8426180
BR_CC19_T_W2	2018	Setting 3	CC	Throat swab	2	EMM1.0	28	ERS6111084	SAMEA8426181
BR_CC19_T_W2_2	2018	Setting 3	CC	Throat swab	3	EMM1.0	28	ERS6111085	SAMEA8426182
BR_CC20_T_W2	2018	Setting 3	CC	Throat swab	2	EMM1.0	28	ERS6111086	SAMEA8426183
BR_CC21_T_W2	2018	Setting 3	CC	Throat swab	2	EMM1.0	28	ERS6111087	SAMEA8426184

*Genomes uploaded to ENA, project PRJEB43915. Timepoints and samples listed in supplementary tables 3-5.

Supplementary Table 9. Genomes from Chalker *et al* (reference 3) used for analysis

Reference	emm	MLST
ERR1359668	EMM1.0	28
ERR1359632	EMM1.0	28
ERR1359833	EMM1.0	28
ERR1359407	EMM1.0	28
ERR1359854	EMM1.0	28
ERR1359412	EMM1.0	28
ERR1359400	EMM1.0	28
ERR1359441	EMM1.0	28
ERR1359627	EMM1.0	28
ERR1359582	EMM1.0	28
ERR1359530	EMM1.0	28
ERR1359469	EMM1.0	28
ERR1359819	EMM1.0	28
ERR1359663	EMM1.0	28
ERR1359370	EMM1.0	28
ERR1359672	EMM1.0	28
ERR1359419	EMM1.0	28
ERR1359337	EMM1.0	28
ERR1359866	EMM1.0	28
ERR1359373	EMM1.0	28
ERR1359723	EMM1.0	28
ERR1359547	EMM1.0	28
ERR1359825	EMM1.0	28
ERR1359454	EMM1.0	28
ERR1359440	EMM1.0	28
ERR1359420	EMM1.0	28
ERR1359732	EMM1.0	28
ERR1359644	EMM1.0	28
ERR1359427	EMM1.0	28
ERR1359613	EMM1.0	28
ERR1359670	EMM1.0	28
ERR1359402	EMM1.0	28
ERR1359385	EMM1.0	28

Reference	emm	MLST
ERR1359861	EMM3.93	315
ERR1359681	EMM3.93	315
ERR1359480	EMM3.93	315
ERR1359372	EMM3.93	315
ERR1359661	EMM3.93	315
ERR1359512	EMM3.93	315
ERR1359363	EMM3.93	315
ERR1359677	EMM3.93	315
ERR1359532	EMM3.93	315
ERR1359820	EMM3.93	315
ERR1359796	EMM3.93	315
ERR1359777	EMM3.93	315
ERR1359858	EMM3.93	315
ERR1359848	EMM3.93	315
ERR1359759	EMM3.93	315
ERR1359485	EMM3.93	315
ERR1359393	EMM3.93	315
ERR1359603	EMM3.93	315
ERR1359657	EMM3.93	315
ERR1359341	EMM3.93	315
ERR1359787	EMM3.93	315
ERR1359699	EMM3.93	315
ERR1359708	EMM3.93	315
ERR1359862	EMM3.93	315
ERR1359731	EMM3.93	315
ERR1359502	EMM3.93	315
ERR1359763	EMM3.93	315
ERR1359601	EMM3.93	315

Reference	emm	MLST
ERR1359656	EMM4.0	39
ERR1359513	EMM4.0	39
ERR1359639	EMM4.0	39
ERR1359353	EMM4.0	39
ERR1359593	EMM4.0	39
ERR1359508	EMM4.0	39
ERR1359808	EMM4.0	39
ERR1359491	EMM4.0	39
ERR1359749	EMM4.0	39
ERR1359783	EMM4.0	39
ERR1359660	EMM4.0	39
ERR1359633	EMM4.0	39
ERR1359450	EMM4.0	39
ERR1359357	EMM4.0	39
ERR1359543	EMM4.0	39
ERR1359424	EMM4.0	39
ERR1359678	EMM4.0	39
ERR1359788	EMM4.0	39
ERR1359446	EMM4.0	39
ERR1359637	EMM4.0	39
ERR1359576	EMM4.0	39
ERR1359765	EMM4.0	39
ERR1359339	EMM4.0	39
ERR1359847	EMM4.0	39
ERR1359662	EMM4.0	39
ERR1359533	EMM4.0	39
ERR1359643	EMM4.0	39

Reference	emm	MLST
ERR1359623	EMM6.0	382
ERR1359694	EMM6.0	382
ERR1359838	EMM6.0	382
ERR1359664	EMM6.0	382
ERR1359461	EMM6.0	382
ERR1359444	EMM6.0	382
ERR1359767	EMM6.0	382
ERR1359756	EMM6.0	382
ERR1359829	EMM6.0	382
ERR1359333	EMM6.0	382
ERR1359426	EMM6.0	382
ERR1359733	EMM6.0	382
ERR1359343	EMM6.0	382
ERR1359867	EMM6.0	382
ERR1359883	EMM6.0	382
ERR1359640	EMM6.0	382
ERR1359735	EMM6.0	382
ERR1359466	EMM6.0	382
ERR1359527	EMM6.0	382

Supplementary Figures

Supplementary Figure S1- Phylogenetic relationship between *S. pyogenes emm*6 strains from outbreak setting 1.

A) Maximum likelihood phylogenetic tree was constructed from recombination-free 270 core SNPs extracted after mapping 17 *S. pyogenes* isolates collected from setting 1 (S1), single isolates from settings 2 and 4 (S2 and S4), and 19 *emm*6.0 scarlet fever isolates from previously published scarlet fever study in the UK (Chalker *et al*. ref 3), indicated with grey circles, to the complete *emm*6 reference strain MGAS10394 (NC_006086). Isolate source is indicated by specific shape at the end of the branch. CC (classroom contact). Settings are colour coded as indicated in the key legend. All study isolates were *emm*6.0, bar one isolate from Setting 1, *emm*6.9, which is separated from the rest of the setting 1 isolates by 40-43 SNPs. **B)** Phylogenetic tree of 16 *emm*6.0 samples from the outbreak in setting 1. Settings and sample source are colour and shape-coded as indicated in panel A. Samples of the same type (source) that are 0 SNP apart are plotted together (one on top of the other). Scale bar indicates nucleotide substitutions per site.

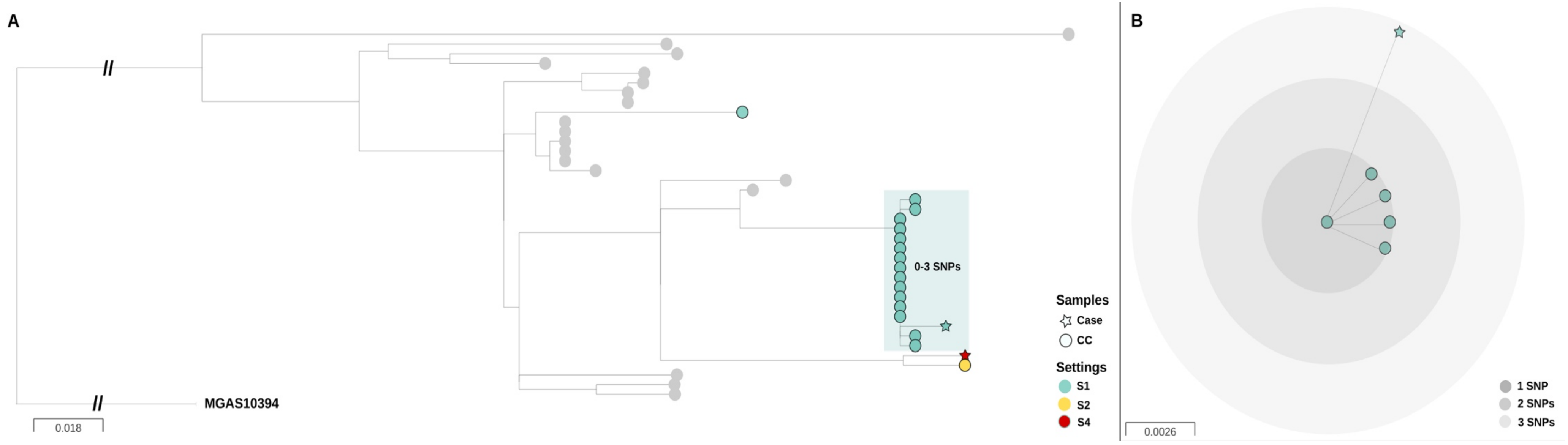

Supplementary Figure S2. **Phylogenetic tree of *S. pyogenes emm*1 strains from outbreak settings 2 and 3.**

A) Maximum likelihood phylogenetic tree constructed from recombination-free 392 core SNPs extracted after mapping 37 *S. pyogenes emm*1.0 isolates collected from settings 2 and 3 (S2, S3), a single isolate from setting 6 (S6); and 33 *emm*1.0 scarlet fever isolates from previously published scarlet fever study in the UK (Chalker *et al*. ref 3), indicated with grey circles, to the complete *emm*1 reference strain MGAS5005 (CP000017). Sample source is indicated by a specific shape at the end of each branch. CC, classroom contact; HHC, household contact. Settings are colour coded as indicated in the key legend. **B)** Phylogenetic tree of 23 *emm*1 isolates from setting 2. **C)** Phylogenetic tree of 10 *emm*1 isolates from setting 3 and two closely related isolates from setting 2. For panels B and C, settings and sample source are colour and shape-coded as indicated in panel A. Isolates from the same sample types that are 0 SNP apart are plotted together; isolates from different sample types with 0 SNP apart are plotted side-by-side to facilitate visualization. Scale bar indicates nucleotide substitutions per site.

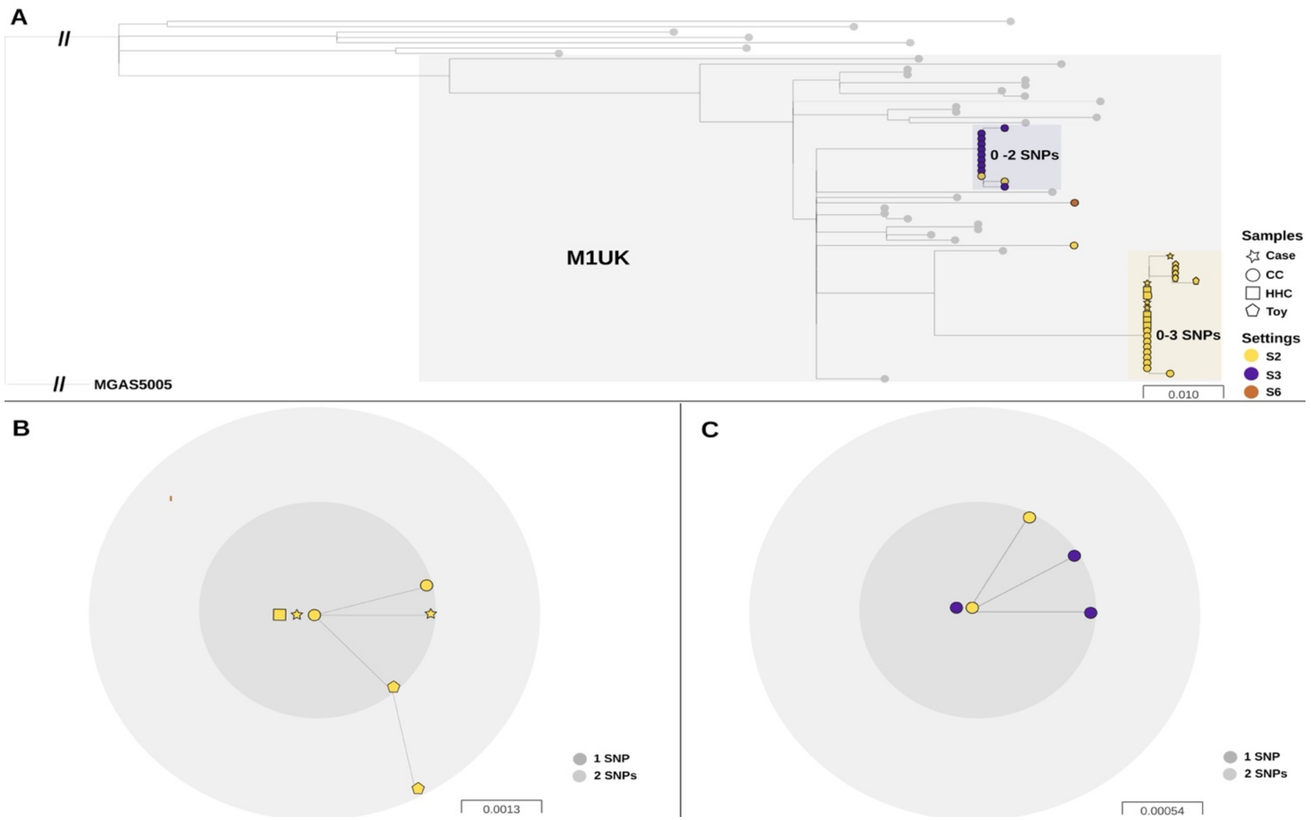

Supplementary Figure S3. **Phylogenetic relationship between *S. pyogenes emm*4 strains from outbreak settings 4 and 5.**

A) Maximum likelihood phylogenetic tree constructed from recombination-free 1,005 core SNPs extracted after mapping 51 *S. pyogenes emm*4.0 isolates collected from settings 4 and 5 (S4, S5); 4 *emm*4.0 isolates from setting 6 (S6); and 27 *emm*4.0 scarlet fever isolates from previously published scarlet fever study in the UK (Chalker *et al*. ref 3), indicated with grey circles to the complete *emm*4 reference strain MGAS10750 (NC_008024). Sample source is indicated by the shape at the end of each branch. CC, classroom contact; Air, environmental settle plate. Settings are colour coded as indicated in the key legend. **B)** Phylogenetic tree of 14 *emm*4.0 isolates from setting 4 outbreak and 4 classroom contact samples from setting 6 that were related with up to 3SNPs difference between the isolates. Settings and sample source are colour and shape-coded as indicated in panel A. Isolates from the same sample types that are 0 SNP apart are plotted together; isolates from different sample types that are 0 SNP apart are plotted side-by-side to facilitate visualization. For setting 5 and setting 4 similar visualization was not performed as no core SNPs differences were detected within the outbreak strains. Scale bar indicates nucleotide substitutions per site.

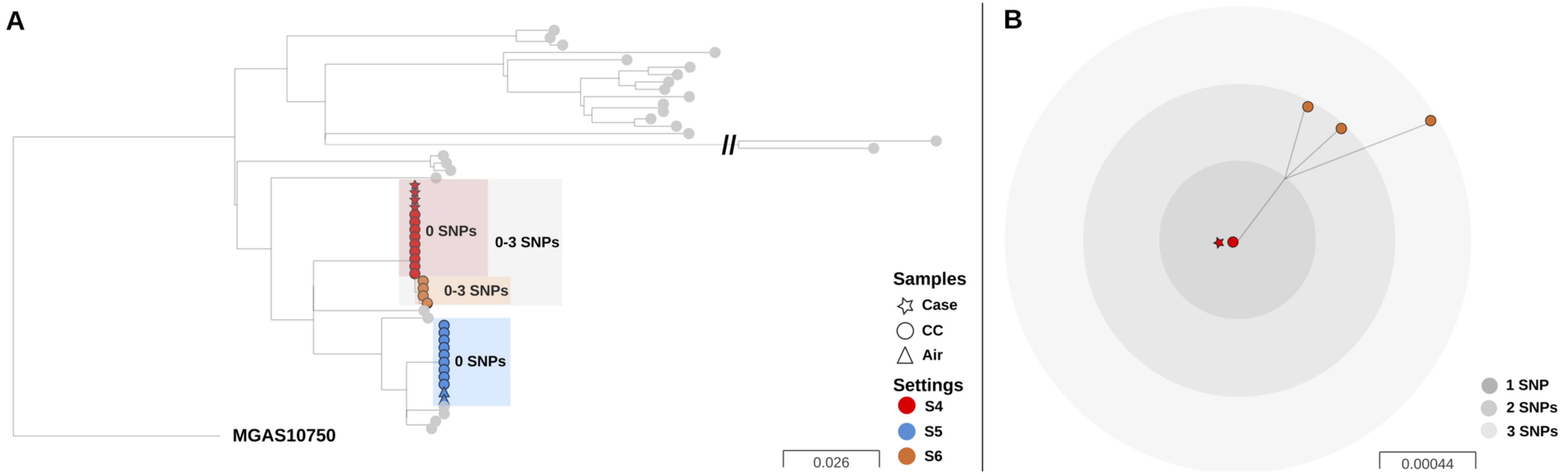

Supplementary Figure S4. **Phylogenetic relationship between *S. pyogenes emm*3 strains from outbreak setting 6.**

A) Maximum likelihood phylogenetic tree constructed from recombination-free 215 core SNPs extracted after mapping 44 *S. pyogenes emm* 3.93 isolates and one 3.143 isolate collected from one setting (S6) and 28 *emm* 3.93 scarlet fever isolates from previously published scarlet fever study in the UK (Chalker *et al*. ref 3), indicated with grey circles, to the complete *emm*3 reference strain MGAS315 (NC_004070). Sample source is indicated by the shape at the end of each branch. CC, classroom contact; HHC, household contact; Air, environmental settle plate. Setting colour coded as indicated in the key legend. **B)** Phylogenetic tree of all 45 *emm*3 isolates from setting 6 outbreak. Settings and sample source are colour and shape-coded as indicated in panel A. Isolates from the same sample types that are 0 SNP apart are plotted together; isolates from different sample types that are 0 SNP apart are plotted side-by-side to facilitate visualization. Scale bar indicates nucleotide substitutions per site.

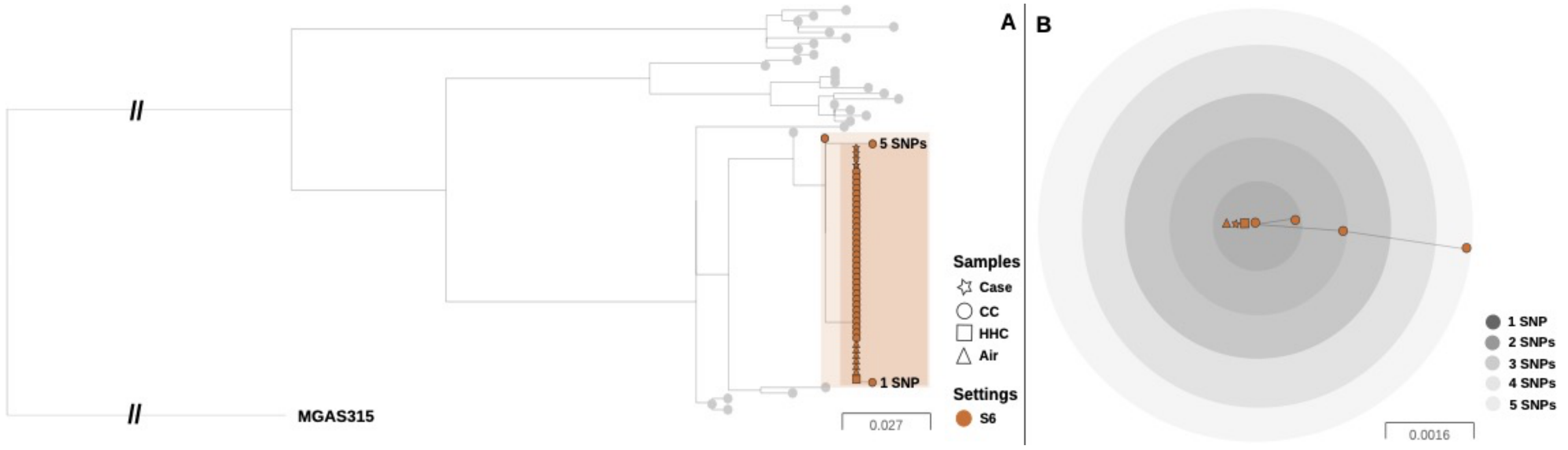

Supplementary Methods

Case definition. Confirmed cases were those with a clinical diagnosis of scarlet fever by a health professional (sore throat, fever, sand-papery rash) and *Streptococcus pyogenes* grown from a throat swab. Probable cases were those with a clinical diagnosis of scarlet fever by a health professional without bacteriological confirmation. Possible cases were those reported by a reliable source (e.g. nursery or school manager), presenting with signs and symptoms consistent with scarlet fever, and a close epidemiological link e.g. household contact of a confirmed case; or attending school where there is a confirmed scarlet fever outbreak.

Recruitment of schools. Confirmed and probable cases of scarlet fever were identified by notifications to local Health Protection Teams. Schools and nurseries were invited to participate if they had two confirmed or probable scarlet fever cases aged 2-8 years from the same class within ten days of each other, with the most recent case arising in the preceding 48h. Locations were excluded if reported cases did not match criteria for cases, if timings did not match inclusion criteria, or if they declined.

Bacteriology and sampling. Swabs from participants were transported in Amies medium (Deltalab, Barcelona, Spain), then plated immediately onto Columbia Blood Agar (CBA, Oxoid, Basingstoke, UK). For cough plates, participants were encouraged to cough onto CBA held at a distance of 20cm from the mouth. CBA plates were incubated in a BSL2 laboratory at 37°C 5% CO_2 overnight then inspected for beta-haemolytic colonies. *S. pyogenes* was identified using Bruker MALDI-ToF Biotyper (Bruker Daltonics, Bremen, Germany). To evaluate presence of *S. pyogenes* DNA in stored culture-negative throat swabs, the *S. pyogenes* housekeeping gene *proS* was amplified from extracted DNA using primers *ProS F* 5′TGAGTTTATTATGAAAGACGGCTATAGTTTC and *ProS R* 5′-AATAGCTTCGTAAGCTTGACGATAATC and copies of *proS* were quantified by comparison with standard concentrations of a plasmid containing a single copy of *proS* as described previously (1). The lower 99% confidence interval of the geometric mean value obtained from 66 culture-positive throat samples from 2019 was used as a cut off (~ 91.6 copies/swab).

In year 1, surface samples (25cm^2) were obtained from frequently touched surfaces in classrooms in week 1 only (20 samples per classroom) using dry cotton swabs moistened in sterile saline. Swabs were placed into 1 ml of sterile saline, and transported to the laboratory where they were diluted using a 10 times serial dilution in sterile phosphate buffered saline to

a dilution of 10^{-6}. Duplicate 50 µl volumes of each dilution were plated on to CBA plates and incubated at 37°C in air supplemented with 5% CO_2 for 48h.

In year 2, air settle plates were used to detect airborne dispersal of *S. pyogenes* using CBA plates placed on horizontal surfaces that were at least 1.5m high such as shelves and cupboards. A pilot study (setting 4) revealed that plates left for 24h were overgrown and un-readable, therefore, for settings 5 and 6, settle plates were left for 2-3h while children were using the classroom. Four plates were used per classroom per time point in settings 4-6, on each of the weeks studied.

Genomic analysis. DNA was extracted from all cultured *S. pyogenes* isolates from overnight cultures and *emm* genotyping was performed according to the protocol of the Centers for Diseases Control and Prevention (www.cdc.gov/ncidod/biotech/strep/protocol_emmtype) followed by pair-end 150bp read length whole genome sequencing on an Illumina HiSeq 2000 platform (Illumina, USA) according to the manufacturer's protocol. In 2019 (settings 4-6) *emm*-typing was performed only from whole genome sequencing data using BLAST+ version 2.2.30 against a specific *S. pyogenes emm*-type database (https://www2.cdc.gov/vaccines/biotech/strepblast.asp). Sequence data have been submitted to the European Nucleotide Archive (ENA - www.ebi.ac.uk/ena) under the accession number PRJEB43915 (Supplementary table 8). Raw reads were trimmed using trimmomatic version 0.36 (2) with the following parameters: trimCrop=N, trimHeadCrop=N, sliding window 5:20, trim leading=3, trim trailing=4, trim min length=55, before any further downstream analysis. The comparative SNP-calling analysis was performed by mapping trimmed reads of 136 *S. pyogenes* isolates to the complete *emm*89 reference sequence H293 (HG316453.2) using Snippy v4.6.0 (https://github.com/tseemann/snippy), with a minimum coverage of 10, minimum fraction of 0.9, and minimum vcf variant call quality of 100.

Gubbins version 2.4.1 (3) was used to identify and remove recombinant regions from the resulting full genome alignment file. A maximum likelihood phylogeny was created from core SNPs using the general time-reversible (GTR) model of nucleotide substitution with the gamma distributed rate heterogeneity implemented in FastTree v2.1.10-4 (4) Phylogenetic trees were visualized using FigTree v1.4.2 (http://tree.bio.ed.ac.uk/software/figtree/) and Microreact (https://microreact.org/showcase) and edited using INKSCAPE (https://inkscape.org/pt/). Genetic diversity within *emm*-types identified as dominating in each setting (*emm 1*, *emm 3*, *emm 4* and *emm 6*) was assessed by comparison with previously sequenced isolates from an earlier UK scarlet fever study (5) (**Supplementary Table 9**) and using *emm*-type specific reference genomes as follows: for *emm1,* MGAS5005 (CP000017); for *emm3,* MGAS315 (NC_004070); for *emm4*, MGAS10750 (NC_008024); for *emm6,*

MGAS10394 (NC_006086). The SNP distance matrix was obtained using snp-dist (https://github.com/tseemann/snp-dists). SNPs identified within each outbreak setting were classified as non-coding, missense or synonymous according to the location in the genome and effect on protein using Snippy. The functional effect of each amino acid substitution was predicted using PROVEAN Protein database (http://provean.jcvi.org/seq_submit.php). *emm*4 sub-lineage classification (M4 “complete” vs M4 “degraded”) was performed as described by Remmington *et al.* 2021 (6), while *emm*1 sub-lineage classification (M1 vs $M1_{UK}$) was performed as described by Lynskey, Jauneikaite *et al.* 2019 (7).

References for supplementary methods

1. Edwards RJ, Pyzio M, Gierula M, Turner CE, Abdul-Salam VB, Sriskandan S. 2018. Proteomic analysis at the sites of clinical infection with invasive *Streptococcus pyogenes*. *Sci Rep*. 2018;8(1):5950. doi:10.1038/s41598-018-24216-2
2. Bolger AM, Lohse M, Usadel B. Trimmomatic: a flexible trimmer for Illumina sequence data, *Bioinformatics*, Volume 30, Issue 15, 1 August 2014, Pages 2114–2120. doi.org/10.1093/bioinformatics/btu170
3. Croucher NJ, Page AJ, Connor TR, Delaney AJ, Keane JA, Bentley SD, Parkhill J, Harris SR. 2015. Rapid phylogenetic analysis of large samples of recombinant bacterial whole genome sequences using Gubbins. Nucleic Acids Res 43:e15.
4. Price, M.N., Dehal, P.S., and Arkin, A.P. (2009) FastTree: Computing Large Minimum-Evolution Trees with Profiles instead of a Distance Matrix. Molecular Biology and Evolution 26:1641-1650, doi:10.1093/molbev/msp077.
5. Chalker V, Jironkin A, Coelho J, Al-Shahib A, Platt S, Kapatai G, Daniel R, Dhami C, Laranjeira M, Chambers T, Guy R, Lamagni T, Harrison T, Chand M, Johnson AP, Underwood A; Scarlet Fever Incident Management Team. Genome analysis following a national increase in Scarlet Fever in England 2014. BMC Genomics. 2017 Mar 10;18(1):224. doi: 10.1186/s12864-017-3603-z. PMID: 28283023; PMCID: PMC5345146
6. Remmington A, Haywood S, Edgar J, Green LR, de Silva T, Turner CE. Cryptic prophages within a *Streptococcus pyogenes* genotype *emm*4 lineage. Microb Genom. 2021 Jan;7(1). doi: 10.1099/mgen.0.000482. Epub 2020 Nov 27. PMID: 33245690.
7. Lynskey NN, Jauneikaite E, Li HK, et al. Emergence of dominant toxigenic M1T1 *Streptococcus pyogenes* clone during increased scarlet fever activity in England: a population-based molecular epidemiological study. Lancet Infect Dis. 2019;19(11):1209-1218. doi:10.1016/S1473-3099(19)30446-3
